# Stereotactic radiosurgery in the management of central nervous system hemangioblastomas: a systematic review and meta-analysis

**DOI:** 10.1007/s10143-025-03454-9

**Published:** 2025-03-17

**Authors:** Amirhossein Zare, Amirhessam Zare, Alireza Soltani Khaboushan, Bardia Hajikarimloo, Jason P. Sheehan

**Affiliations:** 1 Department of Neurosurgery, Tehran University of Medical Sciences, Tehran, Iran; 2 Department of Neurological Surgery, University of Virginia, Charlottesville, VA USA

**Keywords:** Stereotactic radiosurgery, Hemangioblastoma, Von Hippel-Lindau disease, Gamma knife, LINAC, CyberKnife

## Abstract

Central nervous system (CNS) hemangioblastomas are rare, benign vascular tumors occurring sporadically or with von Hippel-Lindau (VHL) disease. While resection remains the primary treatment for symptomatic lesions, stereotactic radiosurgery (SRS) has emerged as an alternative where surgery is not feasible. This research aims to investigate the efficacy and safety of SRS for CNS hemangioblastomas, focusing on long-term outcomes and identifying key prognostic factors. A systematic search was conducted in PubMed, Scopus, Embase, Web of Science, and Cochrane Library till October 4th, 2024. Studies investigating the efficacy of SRS for CNS hemangioblastomas were included. The risk of bias was assessed using the ROBINS-I tool. Meta-analysis, subgroup analysis, and meta-regression were performed using the R programming language. A total of 28 studies with 627 patients and 1761 lesions were included. Our meta-analysis demonstrated pooled overall, 5- and 10-year local tumor control rates of 89% (95% CI: 85–92%), 87% (95% CI: 82–91%), and 80% (95% CI: 63–91%), respectively. Tumor response assessment revealed that 28% (95% CI: 19–40%) of lesions had regression, while 59% (95% CI: 46–70%) remained stable. VHL-associated lesions had a higher control than sporadic tumors at the 5-year follow-up (VHL: 94% (95% CI: 90–97%) vs. Sporadic: 82% (95% CI: 76–86%), *P* < 0.01). Pooled adverse events were 11% (95% CI: 8–15%). Meta-regression identified the female sex as a positive predictor for tumor control and lower adverse event rate (*P* < 0.01 and *P* = 0.02, respectively), while higher marginal and maximum radiation doses correlated with increased adverse events (*P* = 0.02 and *P* = 0.03, respectively). SRS represents a reasonably effective and safe treatment option for CNS hemangioblastomas, particularly in VHL-associated lesions. Patient demographics, VHL status, and tumor characteristics have been analyzed to identify factors potentially impacting treatment outcomes.

**Supplementary Information:**

The online version contains supplementary material available at 10.1007/s10143-025-03454-9.

## Clinical trial number

Not applicable.

**Supplementary Information:**

The online version contains supplementary material available at 10.1007/s10143-025-03454-9.

## Introduction

Central nervous system (CNS) hemangioblastomas are rare, benign vascular neoplasms classified as WHO grade 1 tumors. These lesions manifest either sporadically in 60–75% of cases or as a component of von Hippel-Lindau disease (VHL) in 20–40% of cases [1, 2]. VHL is an autosomal dominant disorder with 95% penetrance by midlife, most commonly presenting with hemangioblastomas, which are the predominant CNS manifestation of the syndrome [3]. Individuals with VHL-associated hemangioblastomas typically experience an earlier disease onset and have a less favorable prognosis due to the formation of multiple neoplasms [4]. The anatomical distribution of CNS hemangioblastomas encompasses the cerebellum (45%), spinal cord (36%), cauda equina (11%), brainstem (7%), and supratentorial region (1%) [1, 2, 5]. These tumors comprise 1–2% of all CNS neoplasms and 8–12% of posterior fossa lesions [2, 5]. While sporadic hemangioblastomas predominantly affect the cerebellum, VHL-associated lesions commonly involve the cerebellum, brainstem, and spinal cord [6]. Despite their benign histology, CNS hemangioblastomas can result in significant neurological morbidity and mortality, depending on the location and number of lesions [7, 8].

Therapeutic options for hemangioblastomas encompass resection, stereotactic radiosurgery (SRS), and active surveillance via serial MRI [1]. Surgical intervention remains the preferred treatment modality for symptomatic hemangioblastomas [9]. However, achieving gross total resection can at times be challenging, particularly in patients with VHL syndrome who frequently develop multiple lesions. In addition, cases involving subtotal resection are usually correlated with a considerable risk of recurrence, especially within the VHL cohort [10, 11]. Given the morbidity associated with repeated resections, ionizing radiation, particularly SRS, has emerged as a viable alternative, either as a primary modality or in conjunction with resection as an adjuvant or salvage therapy. Notably, in VHL cases, SRS mitigates the cumulative surgical burden while enabling the simultaneous management of multiple synchronous or metachronous tumors [11–14].

Despite the increasing application of SRS in managing CNS hemangioblastomas, long-term clinical and radiological outcomes remain poorly defined. While various studies have reported favorable findings, comprehensive data on treatment outcomes are still lacking. This study aims to assess the safety and efficacy of SRS, as well as the impact of key patient and tumor characteristics on outcomes, to clarify its role in the management of CNS hemangioblastomas.

## Materials and methods

### Object

This study followed the Preferred Reporting Items for Systematic Reviews and Meta-Analyses (PRISMA) guidelines [15]. The study protocol has been registered in PROSPERO (ID: CRD42024597988).

### Search strategy

A comprehensive search was conducted across PubMed, Embase, Scopus, Web of Science, and the Cochrane Library till October 4th, 2024, without any language, publication date, or study type restrictions. The search strategy incorporated keywords related to “hemangioblastoma” and “radiosurgery”. Detailed search syntax for all databases is available in the Supplementary Materials (Table S1). Reference lists of included papers were manually searched to identify potentially relevant studies.

### Eligibility criteria

The following PICO framework was utilized to determine the eligibility criteria:

- Population: Individuals diagnosed with CNS hemangioblastomas.
- Intervention: SRS modalities, including gamma knife radiosurgery (GKRS), CyberKnife radiosurgery (CKRS), and linear accelerator (LINAC).
- Comparison: None.
- Outcome: Overall survival (OS), local tumor control (LTC), and adverse events.

Studies were excluded if they were in vivo or in vitro, or if they were case reports, reviews, letters, or book chapters. Additionally, case series with fewer than five patients were excluded due to limited generalizability and an increased risk of publication bias.

### Study selection

Two independent reviewers screened the identified studies based on titles and abstracts, followed by a review of full texts. Discrepancies were resolved through discussion. If multiple studies utilized the same cohort, the most recent study with the largest sample size and longest follow-up period was selected.

### Data extraction

Two reviewers independently extracted relevant data, including study characteristics, baseline characteristics of patients and lesions, radiosurgery details, and clinical and radiological outcomes. Any discrepancies were resolved through discussion or by involving a third reviewer.

### Risk of bias assessment

The Risk of Bias in Non-randomized Studies - of Interventions (ROBINS-I) was used to assess the risk of bias [16]. Two reviewers independently evaluated the studies across the tool’s seven bias domains: bias due to confounding, selection of participants, classification of interventions, deviations from intended intervention, missing data, measurement of outcomes, and selection of reported results. Each domain was rated as low, moderate, serious, critical risk, or no information. Disagreements were resolved through discussion. The overall risk of bias was determined by the highest risk level across any domain. The results were visualized using a traffic light plot generated with the “robvis” package in R software [17].

### Statistical analysis

Values reported in median and range were converted to mean and standard deviation (SD) [18]. Random-effect meta-analysis was conducted using the restricted maximum-likelihood (REML) estimation weighting each of the studies by their inverse variance. Meta-regression was performed to determine sources of heterogeneity. Additionally, subgroup analyses were conducted to explore the influence of various factors on outcomes, including patient demographics, VHL status, and tumor characteristics. Leave-one-out analysis was performed to determine the impact of individual studies on the overall effect estimates. Publication bias was evaluated through visual inspection of funnel plot asymmetry and quantified using Egger’s regression test. A P-value < 0.05 was considered statistically significant. All statistical analyses were conducted in R (version 4.1.2, R Foundation for Statistical Computing) using the “meta” and “metafor” packages.

## Results

### Study selection

**Fig. 1:**
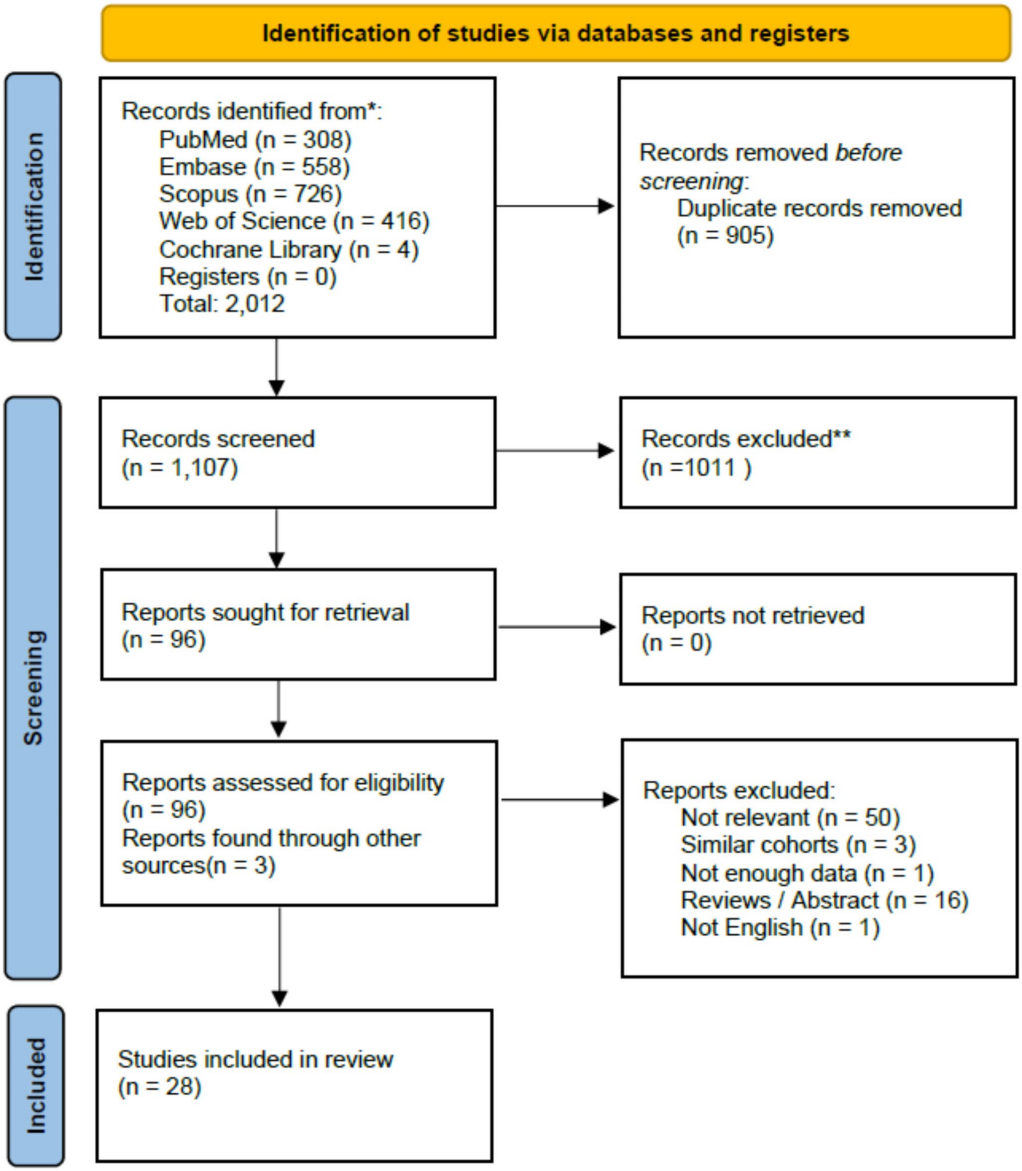
PRISMA flowchart of screening and selection process

Our search strategy retrieved 2012 studies across multiple databases, of which 905 publications were identified as duplicates and removed subsequently. Title and abstract screening excluded 1011 studies. The full text of the remaining 96 publications was screened, and a total of 32 studies met our eligibility criteria. Three studies [19–21] had their outcomes later incorporated into a publication [9] with expanded sample sizes and additional outcome measures, leading us to exclude them. One excluded by not reporting the outcomes of interest [22]. No article was added through reference checking. The details of the study selection process are summarized in the PRISMA flow diagram depicted in Fig. 1.

### Quality assessment

Of the 28 studies included, 25 were assessed as having a moderate risk of bias, while 3 were determined to have a serious risk of bias (Supplementary Fig. 1). The primary source of risk across studies was bias in the selection of participants, with three studies showing a serious risk in this domain. Restrictive inclusion and exclusion criteria were the primary cause of this selection bias, which may impact the generalizability of findings. Moreover, confounding bias and missing data bias were other notable sources of risk of bias. These biases were primarily due to inadequate adjustment for relevant covariates and inconsistencies in follow-up, respectively.

### Baseline characteristics

Characteristics of the included studies are summarized in Table 1. The 28 included studies were published between 1996 and 2023. Two studies employed a prospective design, while 26 used a retrospective approach. The studies were conducted in 11 countries, with the USA accounting for 50%. The mean follow-up of patients after radiosurgery ranged from 15 to 102 months. The included studies comprised 627 patients with 1761 lesions. Female patients accounted for 44% of the patients. The mean age of patients in the included studies ranged from 26.4 to 56.6. Among patients for whom disease status was reported, 352 (59%) cases had VHL syndrome. VHL-associated lesions made up the majority (74%) of the lesions with defined status. Of the lesions with specified locations, 1556 (89%) were located in the intracranial compartment, with the remaining lesions located in the spinal region. The distribution of intracranial lesions revealed a predominance in the cerebellar areas (*n* = 1184), followed by brainstem localization (*n* = 152) and supratentorial involvement (*n* = 61).

**Table 1:** Summary of characteristics of 28 included studies

Study	Modality	Country	Design	No. of Pts.	No. of lesions	Female	Mean age	VHL Pts.	VHL lesions	Intracranial location	Spinal location	Mean Tumor Volume	Follow-up after SRS (months)	Marg. dose (Gy)	Max. dose (Gy)	Prior surgery	Risk of bias
Carrete 2023 [13]	NA	USA	Retro.	27	123	33%	41.1	NA	92%	123	0	0.56	77.6	18	28.3	NA	Moderate
Palmer 2023 [23]	CKRS	UK	Retro.	10	14	30%	47.3	90%	NA	NA	NA	NA	15.4	9.6	14.3	10%	Moderate
Yoo 2023 [12]	CKRS	USA	Retro.	35	135	51%	39.8	80%	91%	93	42	1.4	57	NA	NA	83%	Moderate
Cvek 2022 [24]	CKRS	Czech Republic	Retro.	5	18	40%	30.5	NA	NA	0	18	1.07	60	NA	32.5	NA	Moderate
Zibly 2020 [25]	LINAC	Israel	Retro.	14	23	36%	43	64%	48%	23	0	1.06	89	15.7	21.7	NA	Moderate
Liebenow 2019 [26]	GKRS	USA	Retro.	15	101	67%	44.1	67%	95%	101	0	2.31	5.4	NA	NA	67%	Moderate
Pan 2017 [27]	CKRS	USA	Retro.	28	46	50%	47.7	50%	48%	0	46	1.89	54.3	21.6	28.1	71%	Moderate
Goyal 2016 [28]	GKRS	India	Retro.	10	27	30%	32.9	80%	93%	25	2	1.64	48	29.9	NA	60%	Serious
Silva 2016 [29]	GKRS	USA	Retro.	12	20	50%	54.3	33%	55%	20	0	4.48	78	21.7	38	92%	Moderate
Kano 2015 [9]	GKRS	Japan	Retro.	186	517	49%	49.2	43%	65%	517	0	1.74	60	16.7	29.5	84%	Moderate
Hanakita 2014 [30]	GKRS	Japan	Retro.	21	97	48%	46.2	67%	93%	97	0	2.41	107	17.5	38.2	95%	Moderate
Puataweepong 2014 [31]	LINAC + CKRS	Thailand	Prosp.	14	56	29%	45.7	64%	NA	56	0	5.2	37	20.5	NA	NA	Moderate
Selch 2012 [32]	LINAC	USA	Retro.	9	20	78%	46.5	56%	80%	0	20	1.52	51	NA	14.3	NA	Moderate
Asthagiri 2010 [14]	GKRS + LINAC	USA	Prosp.	20	44	50%	37.5	100%	100%	44	0	0.5	102	18.9	28.9	75%	Moderate
Chang 2010 [33]	CKRS	Korea	Retro.	5	8	80%	26.4	NA	NA	0	8	0.25	49	NA	NA	NA	Moderate
Daly 2010 [34]	CKRS	USA	Retro.	19	27	47%	35.4	74%	NA	0	27	2.54	NA	NA	NA	NA	Serious
Karabagli 2010 [35]	GKRS	Turkey	Retro.	13	34	46%	33.3	54%	82%	34	0	0.91	50.2	NA	NA	NA	Moderate
Moss 2009 [36]	LINAC + CKRS	USA	Retro.	28	82	36%	39.5	89%	96%	66	16	1.8	76	23.4	NA	93%	Moderate
Park 2005 [37]	GKRS	Korea	Retro.	9	84	44%	37.5	56%	11%	84	0	0.82	52	16.6	NA	89%	Moderate
Tago 2005 [38]	GKRS	Japan	Retro.	13	38	23%	43.4	54%	NA	37	1	1.57	58	18.4	39	100%	Moderate
Wang 2005 [39]	GKRS	China	Retro.	35	93	20%	36	60%	NA	93	0	NA	66	17.2	35.6	100%	Moderate
Rajaraman 2004 [40]	GKRS	UK	Retro.	14	30	36%	35	100%	100%	30	0	2.1	34	19.5	NA	100%	Moderate
Ryu 2003 [41]	CKRS	USA	Retro.	5	7	40%	56.6	80%	NA	0	7	NA	NA	NA	27	60%	Moderate
Jawahar 2000 [42]	GKRS	USA	Retro.	27	29	NA	32	59%	NA	29	0	3.2	NA	16.1	32.5	96%	Moderate
Chang 1998 [43]	LINAC + CKRS	USA	Retro.	13	29	23%	40	100%	100%	27	2	1.6	43	23.2	NA	85%	Moderate
Georg 1997 [44]	GKRS	USA	Retro.	8	10	50%	49	38%	NA	9	1	4.02	NA	16.4	33.5	100%	Serious
Niemela 1996 [45]	GKRS	Finland	Retro.	10	11	50%	45	40%	55%	11	0	NA	43	20.7	37.6	80%	Moderate
Patrice 1996 [46]	LINAC	USA	Retro.	22	38	41%	44.7	41%	61%	37	1	3.49	24	15.7	27.8	NA	Moderate

Pts.: patients, CKRS: CyberKnife Radiosurgery, GKRS: Gamma Knife Radiosurgery, LINAC: Linear Accelerator, Retro.: Retrospective, Prosp.: Prospective, NA: Not Available, Marg. dose: Marginal dose, Max. dose: Maximum dose

GKRS was the most commonly used modality (*n* = 13 studies), followed by CKRS (*n* = 7 studies) and LINAC (*n* = 3 studies). Four studies used more than one modality. The mean marginal dose ranged from 9.6 to 29.9 Gy in 20 studies, with a median of 18.2 Gy. The maximum dose fell in the range of 14.3 to 39 Gy, with a median of 29.5 Gy (*n* = 17 studies).

### Radiologic outcomes

#### Temporal Local Tumor Control (LTC)

Eighteen studies reported 1- and 3-year LTC rates for a total of 1319 and 1387 lesions, respectively. The pooled 1- and 3-year LTC was 96% (95% CI: 94–97%) and 89% (95% CI: 84–92%), respectively (Table 2). Meta-regression showed older age associated with a higher 1-year LTC (*P* < 0.01). Older age (*P* = 0.03) and female sex (*P* < 0.01) were associated with higher 3-year LTC rates. Meta-regression results for the main outcomes are summarized in Table 3. Full meta-regression results are detailed in Supplementary Table S2.

**Table 2:** Summary of meta-analysis results for different outcomes

Outcome	No. of Studies	No. of Lesions/Patients	Pooled Estimate (95% CI)	I²	τ²	H
1-Year LTC	18	1319	0.96 (0.94–0.97)	0.33	0.32	1.22
3-Year LTC	18	1387	0.89 (0.84–0.92)	0.68	0.44	1.78
5-Year LTC	19	1522	0.87 (0.82–0.91)	0.72	0.45	1.90
10-Year LTC	4	704	0.80 (0.63–0.91)	0.79	0.69	2.16
5-Year OS	10	360	0.89 (0.81–0.94)	0.36	0.32	1.25
Overall LTC	25	1592	0.89 (0.85–0.92)	0.58	0.38	1.55
Stable Tumor	16	1163	0.59 (0.46–0.70)	0.84	0.79	2.48
Tumor Regression	16	1163	0.28 (0.19–0.40)	0.85	0.83	2.54
Symptom control	8	156	0.84 (0.76–0.89)	0.03	0	1.01
Post-SRS Surgical Resection	17	1335	0.08 (0.05–0.11)	0.55	0.32	1.49
Adverse Event Rate	21	521	0.11 (0.08–0.15)	0.02	0.10	1.00
Radiation Necrosis	13	207	0.09 (0.05–0.15)	0	0.17	1.00

**Table 3:** Meta-regression results for main outcomes

Outcome	Covariate	No. of Studies	Estimate	Estimate SE	*P* Value	*R*²	I²	τ²
1-Year LTC	Female	17	0.583	1.53	0.703	0	39.63	0.33
	Marginal dose	13	0.053	0.106	0.621	0	46.12	0.37
	Maximum dose	11	0.029	0.042	0.489	0	50	0.4
	Age	18	0.092	0.03	0.002	92.07	4.51	0.02
	No. of lesions per patient	18	0.138	0.16	0.388	0	38.5	0.33
	Tumor volume	16	0.249	0.214	0.243	11.86	35.68	0.32
	VHL associated lesions	11	-2.953	1.497	0.048	67.16	18.42	0.11
	Publication year	18	-0.003	0.037	0.928	0	39.29	0.36
3-Year LTC	Female	17	3.201	1.179	0.007	60.46	48.23	0.17
	Marginal dose	13	0.021	0.095	0.824	0	74.07	0.38
	Maximum dose	10	-0.002	0.041	0.970	0	63.47	0.23
	Age	18	0.058	0.027	0.032	49.41	53.56	0.22
	No. of lesions per patient	18	0.221	0.147	0.135	10.57	69.42	0.38
	Tumor volume	17	-0.17	0.173	0.326	0	72.66	0.5
	VHL associated lesions	12	-0.772	1.607	0.631	0	75.24	0.61
	Publication year	18	0.039	0.031	0.205	12.71	67.96	0.37
5-Year LTC	Female	18	3.638	0.947	< 0.001	81.7	36.13	0.08
	Marginal dose	13	0.031	0.093	0.740	0	76.53	0.37
	Maximum dose	10	-0.007	0.044	0.865	0	75.23	0.35
	Age	19	0.05	0.029	0.078	32.49	65.22	0.29
	No. of lesions per patient	19	0.237	0.133	0.076	22.46	71.15	0.34
	Tumor volume	18	-0.129	0.157	0.412	0	73.31	0.42
	VHL associated lesions	13	-0.647	1.442	0.654	0	75.37	0.49
	Publication year	19	0.04	0.026	0.116	25.67	69.57	0.32
5-Year OS	Female	9	1.459	3.028	0.630	0	17.1	0.18
	Marginal dose	7	0.111	0.206	0.590	0	50.19	0.4
	Maximum dose	8	-0.122	0.072	0.090	30.13	24.88	0.17
	Age	10	0.05	0.053	0.339	0.25	31.55	0.41
	No. of lesions per patient	10	0.507	0.188	0.007	100	0	0
	Tumor volume	9	-0.836	0.302	0.006	100	0	0
	VHL associated lesions	7	3.425	2.581	0.184	77.64	10.81	0.09
	Publication year	10	0.08	0.025	0.001	100	0	0
Adverse Event Rate	Female	21	-3.503	1.515	0.021	100	0	0
	Marginal dose	16	0.134	0.056	0.017	100	0	0
	Maximum dose	13	0.069	0.032	0.033	100	0	0
	Age	21	-0.005	0.037	0.89	0	16.41	0.12
	No. of lesions per patient	21	0.008	0.129	0.949	0	17.62	0.12
	Tumor volume	19	0.005	0.21	0.982	0	20.8	0.15
	VHL associated lesions	16	1.075	0.905	0.235	57.22	8.85	0.05
	Publication year	21	-0.041	0.021	0.055	80.65	3.38	0.02
Radiation Necrosis	Female	13	-4.582	2.234	0.04	100	0	0
	Marginal dose	9	0.125	0.075	0.094	100	0	0
	Maximum dose	8	0.022	0.054	0.681	0	0	0
	Age	13	-0.05	0.054	0.353	15.89	12.85	0.15
	No. of lesions per patient	13	0.292	0.401	0.467	0	18.89	0.24
	Tumor volume	11	-0.086	0.36	0.81	0	20.56	0.24
	VHL associated lesions	11	3.795	1.564	0.015	100	0	0
	Publication year	13	-0.042	0.03	0.159	99.54	0.07	0

**Fig. 2:**
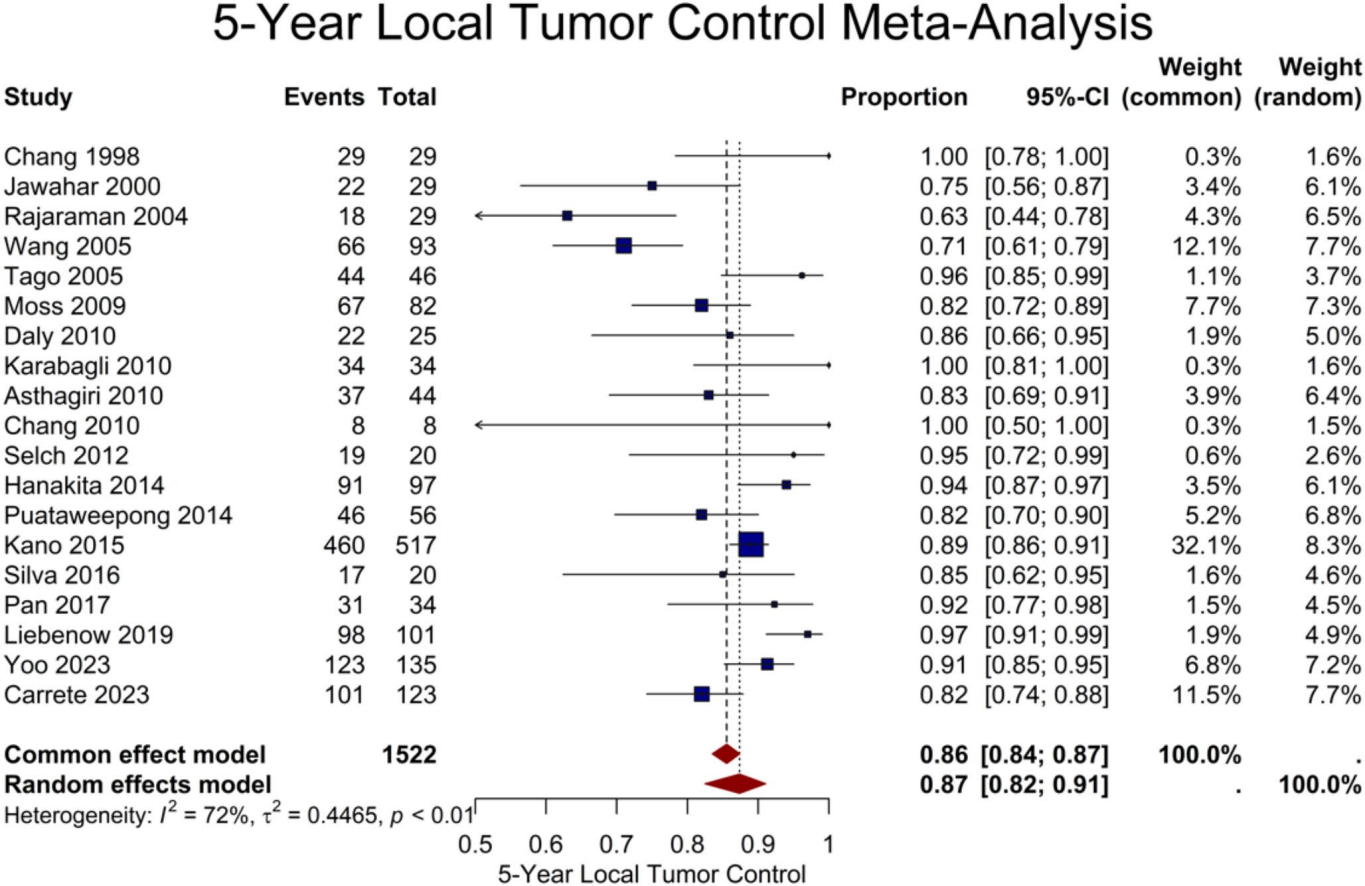
Forest plot showing the 5-year local tumor control rate

**Fig. 3:**
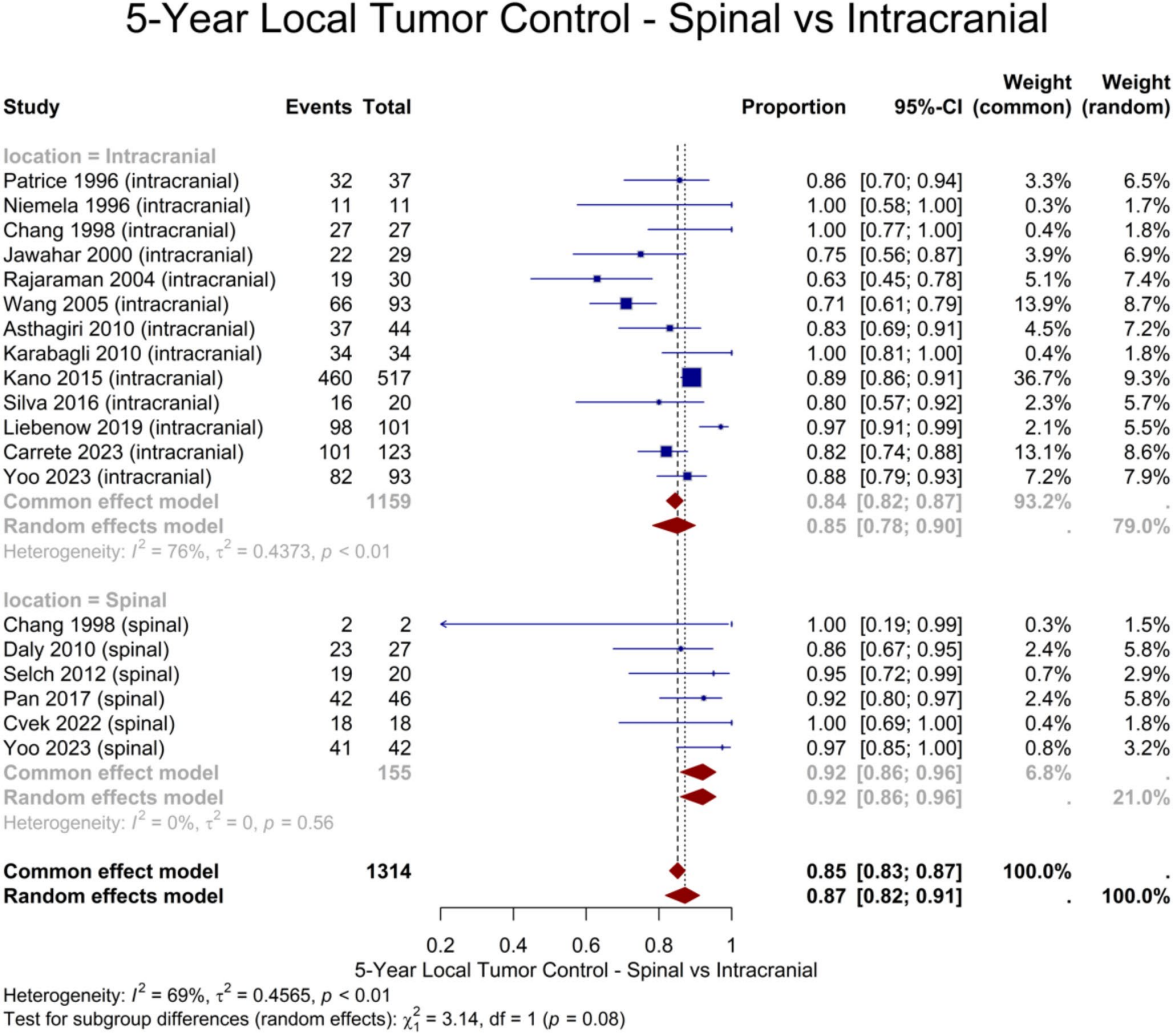
Forest plot showing the subgroup analysis on 5-year LTC in intracranial and spinal lesions

Regarding long-term results, 19 studies provided 5-year LTC for 1522 lesions, resulting in a pooled estimate of 87% (95% CI: 82–91%) (Fig. 2). Age and female gender were shown to be potential sources of heterogeneity, with near significant (*P* = 0.08) and significant association (*P* < 0.001) with a higher 5-year LTC rate, respectively. A 10-year LTC rate was reported in 4 studies, encompassing 704 lesions, with a pooled rate of 80% (95% CI: 63–91%). A summary of the meta-analysis results of all outcomes is presented in Table 2. To investigate potential differences between spinal and intracranial lesions, we conducted subgroup analyses of 19 cohorts, comprising 1314 lesions, with isolated spinal or intracranial lesions. The results revealed no significant differences in 1-, 3-, and 5-year LTC (*P* = 0.36, *P* = 0.30, and *P* = 0.08, respectively) (Fig. 3). Table 4 presents detailed results of the subgroup analyses of spinal vs. intracranial cohorts.

**Table 4:** Details of subgroup analyses of isolated cohorts

Analyzed Cohorts	Outcome	Subgroup	No. of Studies	No. of Lesions	Pooled Estimate (95% CI)	*P*-Value for Subgroup Difference
VHL vs. Sporadic Cohorts	1-Year LTC	Sporadic	6	214	0.93 (0.79–0.98)	0.17
		VHL	8	677	0.97 (0.94–0.99)	
	3-Year LTC	Sporadic	6	214	0.87 (0.81–0.91)	< 0.001
		VHL	8	677	0.94 (0.92–0.96)	
	5-Year LTC	Sporadic	6	217	0.82 (0.76–0.86)	< 0.001
		VHL	8	751	0.94 (0.90–0.97)	
	Overall LTC	Sporadic	10	301	0.85 (0.72–0.92)	0.25
		VHL	12	880	0.91 (0.85–0.94)	
Spinal vs. Intracranial Cohorts	1-Year LTC	Intracranial	13	1159	0.96 (0.93–0.97)	0.36
		Spinal	6	155	0.94 (0.87–0.97)	
	3-Year LTC	Intracranial	13	1159	0.88 (0.82–0.92)	0.30
		Spinal	6	155	0.92 (0.86–0.96)	
	5-Year LTC	Intracranial	13	1159	0.85 (0.78–0.90)	0.08
		Spinal	6	155	0.92 (0.86–0.96)	

#### Overall Local Tumor Control (LTC)

**Fig. 4:**
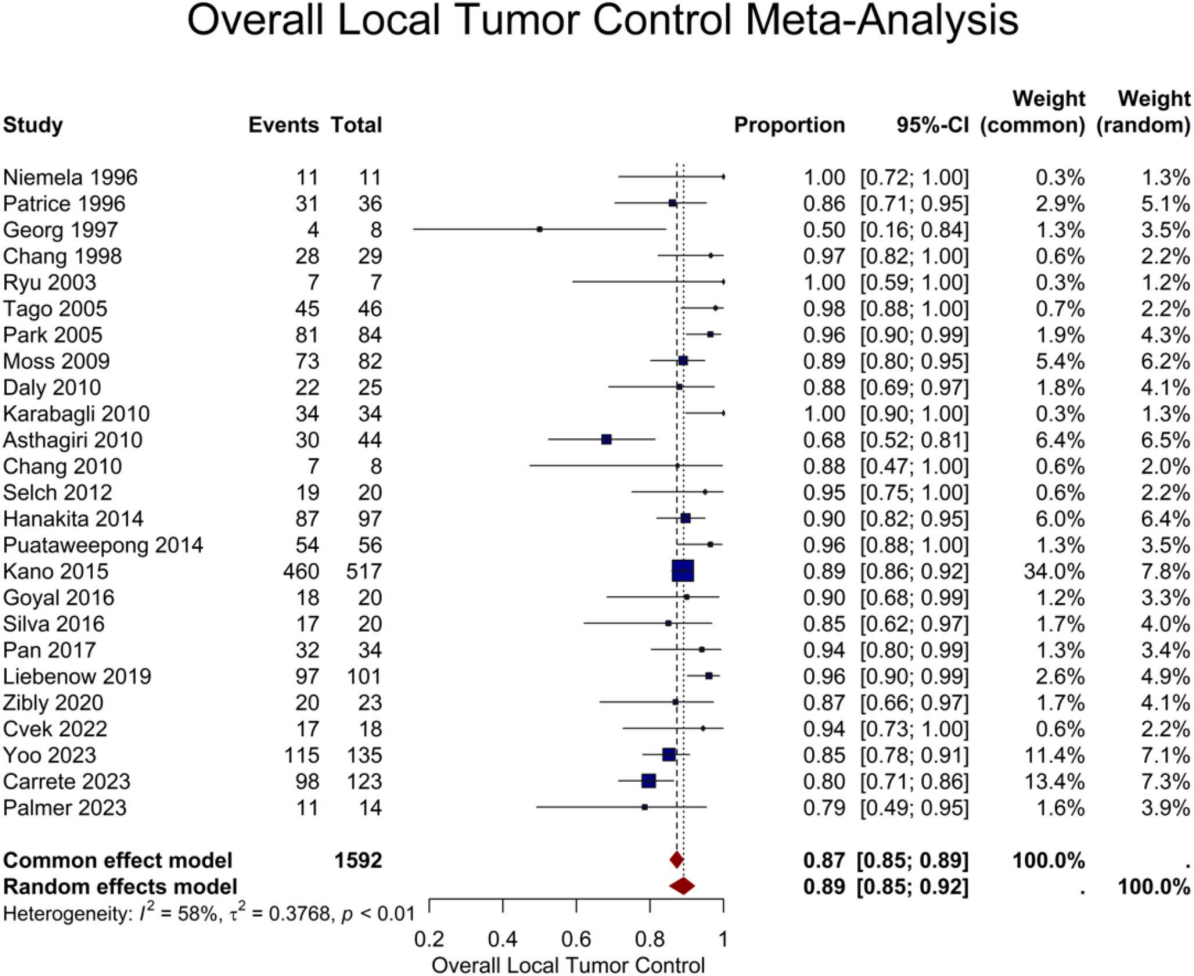
Forest plot demonstrating the overall local tumor control rate

The overall LTC rate was reported in 25 studies for a total number of 1592 lesions, yielding a pooled rate of 89% (95% CI: 85–92%) (Fig. 4). The number of lesions per patient explained some of the heterogeneity observed (*P* = 0.03). In the 16 studies that provided more detailed classifications of tumor response in a total of 1163 lesions, a 28% (95% CI: 19–40%) pooled proportion of lesions showed evidence of tumor regression, and 59% (95% CI: 46–70%) of them remained stable by the end of the radiologic follow-up (Table 2).

#### VHL-Associated vs. Sporadic LTC

**Fig. 5:**
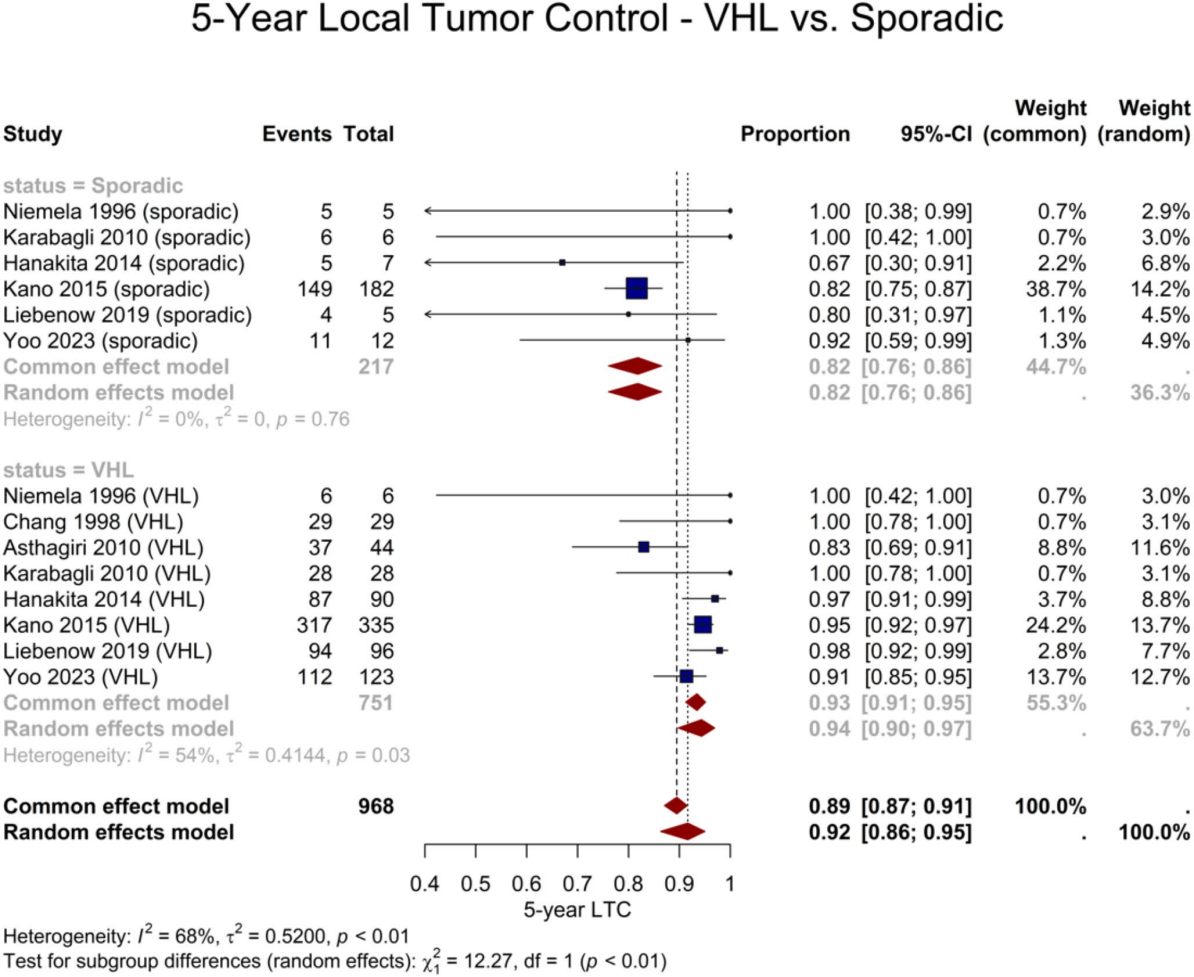
Forest plot demonstrating the difference in 5-year LTC between sporadic and VHL-associated lesions

Twelve studies provided LTC outcomes, differentiating between VHL-associated hemangioblastomas and sporadic lesions. Meta-analysis on 14 cohorts revealed a significantly higher rate of 3- and 5-year LTC for tumors associated with VHL disease (both *P* < 0.001). A total of 751 VHL-associated lesions yielded a pooled 5-year LTC of 94% (95% CI: 90–97%), compared to the rate of 82% (95% CI: 76–86%) in 217 sporadic lesions (Fig. 5).

**Fig. 6:**
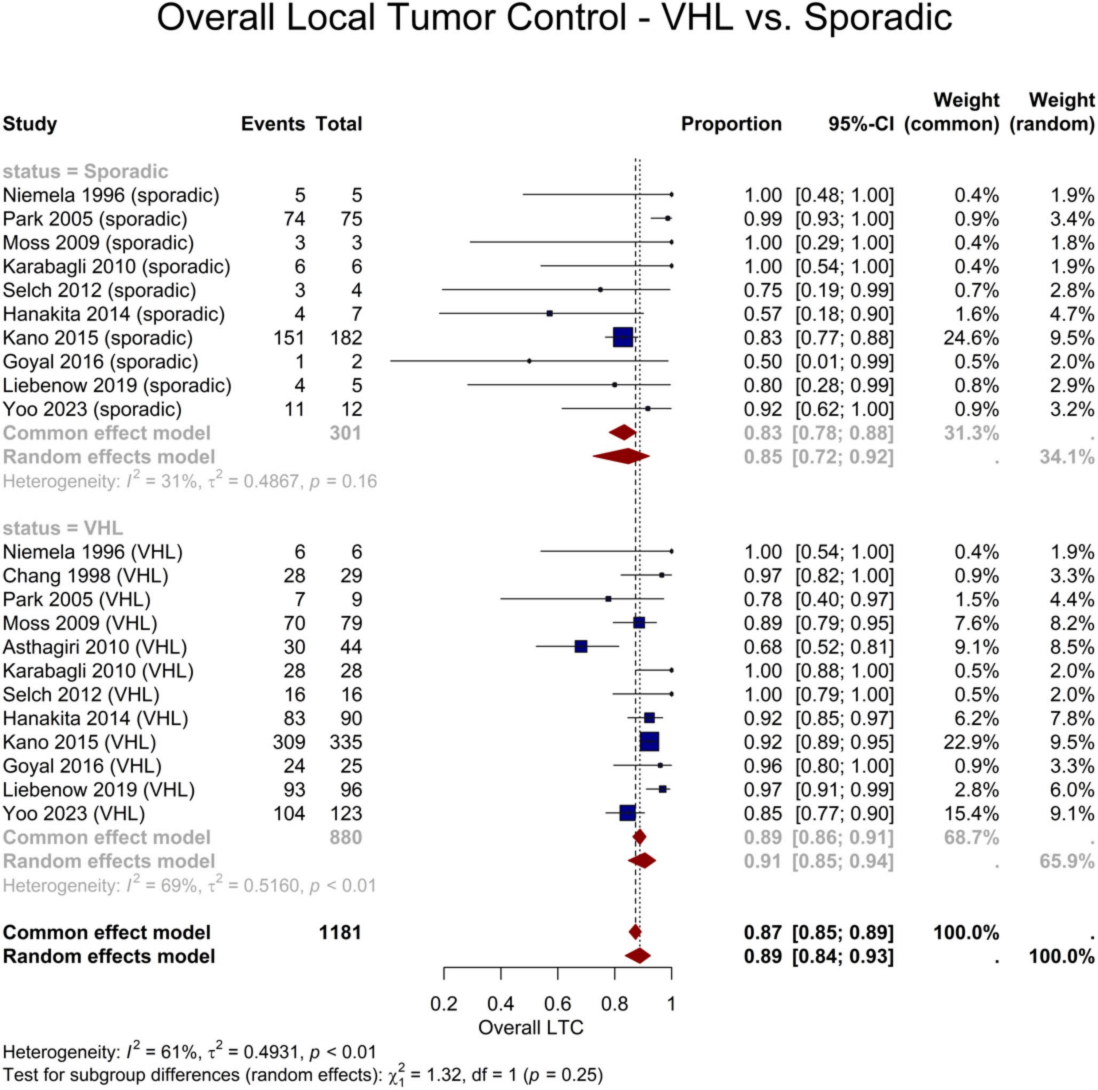
Forest plot demonstrating the difference in overall LTC between sporadic and VHL-associated lesions

The overall LTC rate was provided for 22 cohorts, encompassing a total of 1181 lesions. The pooled overall LTC rate was 91% (95% CI: 85–94%) for the VHL-associated subgroup and 85% (95% CI: 72–92%) for the sporadic subgroup. However, the subgroups’ difference was insignificant (*P* = 0.25) (Fig. 6). Table 4 presents detailed results of the subgroup analyses on VHL vs. sporadic cohorts.

### Clinical outcomes

#### Overall survival

The 5-year OS rate was reported in 10 studies comprising a total of 360 patients, with a pooled rate of 89% (95% CI: 81–94%). Meta-regression analysis revealed significant associations, with 5-year OS showing a positive correlation with year of publication and a negative correlation with mean tumor volume (both *P* < 0.01). Meta-regression results for the main outcomes are summarized in Table 3.

#### Symptom Control

**Fig. 7:**
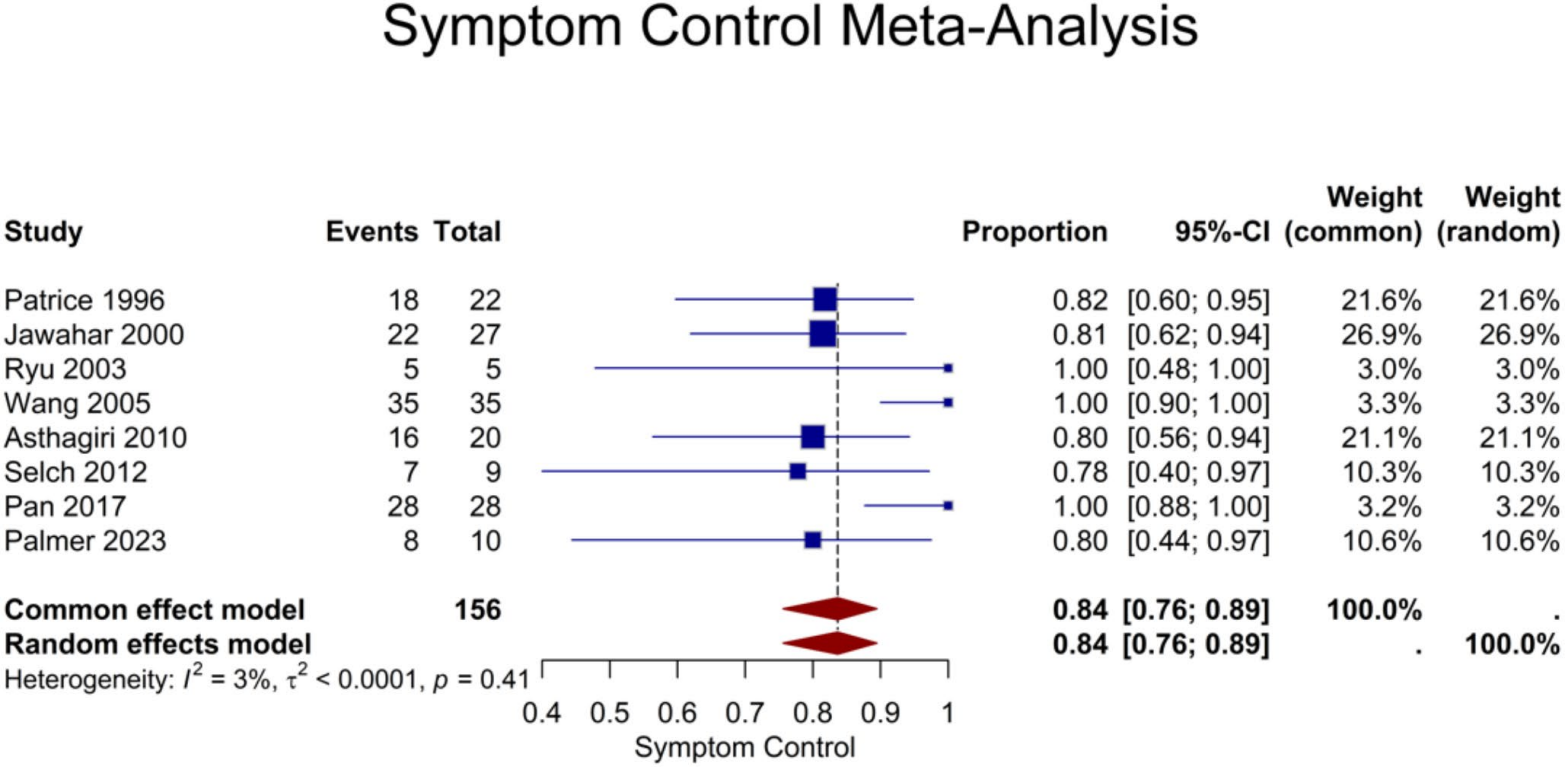
Forest plot showing the proportion of patients with stable or improved neurological symptoms

Eight studies reported outcomes of neurological symptom control. Of the total 156 patients, a pooled proportion of 84% (95% CI: 76–89%) demonstrated either stable or improved neurological symptoms following the procedure (Fig. 7).

### Adverse Radiation Events (AREs)

**Fig. 8:**
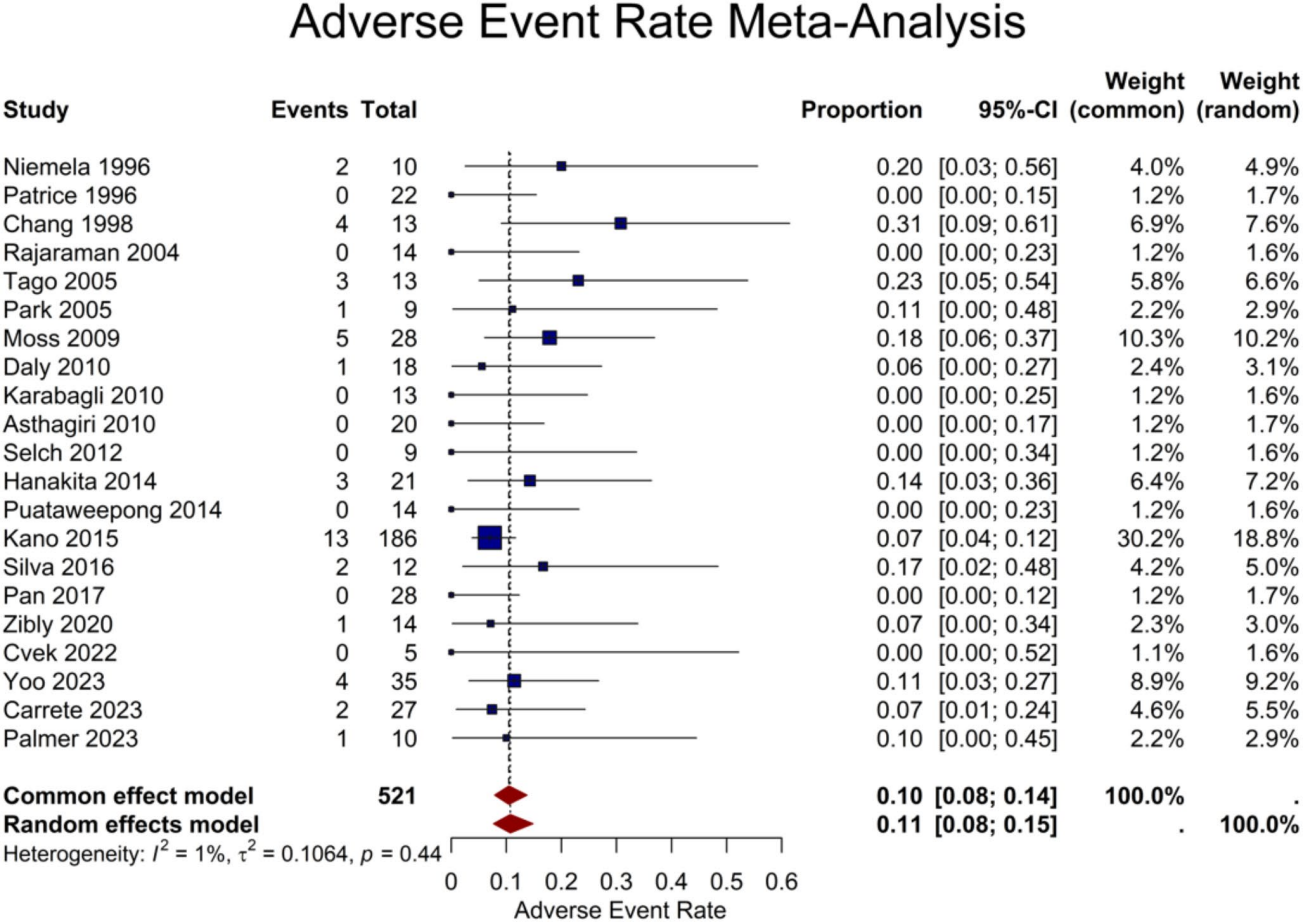
Forest plot showing the adverse event rate

Twenty-one studies provided adverse event rate for a total of 521 patients. The pooled rate was calculated to be 11% (95% CI: 8–15%) (Fig. 8). Meta-regression revealed a significant association of adverse events with higher marginal (*P* = 0.02) and maximum doses (*P* = 0.03). Subsequent subgroup analysis aligned with these results, showing significant differences between various marginal (< 20 vs. ≥ 20 Gy) and maximal dose (< 30 vs. ≥ 30 Gy) groups (*P* = 0.04 and *P* = 0.01, respectively). Detailed outcomes of subgroup analysis based on different variables are provided in Supplementary Table S3. Additionally, female sex was correlated with a lower adverse event rate (*P* = 0.02). The more recent year of publication also revealed a near-significant association with reduced adverse event rate (*P* = 0.054). Being a clinically significant adverse radiation event, radiation necrosis rate was reported in 13 studies, encompassing a total of 207 patients, with a pooled rate of 9% (95% CI: 5–15%).

### Sensitivity analysis

Sensitivity analysis was performed utilizing the leave-one-out analysis to assess the robustness of the results. The robustness of 1-, 3-, 5-, and 10-year and overall LTC results was confirmed by the sensitivity analysis (Supplementary Figs. 2–6). Sensitivity analysis demonstrated that the results for stable tumor and tumor regression rates were considerably altered when Kano 2015 was omitted (Supplementary Figs. 7–8). No outlier was detected by the sensitivity analysis of 5-year OS, symptom control, and post-SRS surgical resection (Supplementary Figs. 9–11). Sensitivity analysis of adverse event rate and radiation necrosis rates demonstrated the robustness of the results as well (Supplementary Figs. 12–13).

### Publication bias

No evidence of funnel plot asymmetry was observed for the 1-, 3-, and 5-year LTC rates, as well as for stable tumor and tumor regression rates, as confirmed by Egger’s test P-values exceeding 0.05 (Supplementary Figs. 14–19). Similarly, no asymmetry was detected in the funnel plots for the 5-year OS, post-SRS resection, and adverse event rates, confirmed by Egger’s test P-values > 0.05 (Supplementary Figs. 20–22). Regarding the radiation necrosis rate, there was significant publication bias revealed by Egger’s test (*P* < 0.001), indicating a tendency to underreport radiation necrosis incidence in studies with smaller sample sizes or less favorable outcomes (Supplementary Fig. 23). After correction using the trim-and-fill method, the adjusted pooled rate was determined to be 13% (95% CI: 8–19%). Visual inspection of the funnel plot did not reveal substantial publication bias regarding the 10-year LTC and symptom control (Supplementary Figs. 24–25).

## Discussion

### Summary

This systematic review comprehensively evaluated the current literature on the utilization of SRS for CNS hemangioblastomas, regardless of its treatment line. Evidence consistently confirms the safety and efficacy of SRS for CNS hemangioblastomas. An overall LTC rate of 89%, concurrent with an adverse event rate of 11%, was observed. Following SRS, 28% of lesions experienced volume regression, and 59% remained stable, indicating significant efficacy of the intervention. Female sex was associated with better tumor control by SRS. Additionally, the incidence of adverse events showed a significant positive correlation with marginal and maximum radiation doses and a negative correlation with female sex and the publication year of the included studies.

This study provides insights into the management of CNS hemangioblastomas, particularly in VHL-associated cases, emphasizing the balance between tumor control and minimizing treatment-related morbidity. For asymptomatic lesions in VHL patients, active surveillance with serial MRI is generally recommended [1]. When intervention is required, the choice between surgical resection and stereotactic radiosurgery (SRS) should be individualized based on patient demographics, tumor characteristics, multiplicity of tumors, symptomatology, goals of care, and the patient’s overall condition.

Resection is typically preferred for symptomatic and surgically accessible tumors, as it often provides immediate symptom relief and long-term control [47, 48]. However, when resection is not feasible due to tumor location or the patient’s goals of care or comorbidities, SRS serves as a very reasonable alternative, offering effective tumor control in appropriately selected patients [12–14]. Additionally, SRS can be considered as an adjuvant or salvage therapy for patients with incompletely resected or recurrent lesions [9, 12–14, 22, 49].

### Efficacy

Several studies have confirmed the efficacy of SRS in achieving local tumor control of hemangioblastomas. Pan et al., in a systematic review and meta-analysis, documented a 5-year progression-free survival rate of 88.43%. Their analyses, which examined variables including VHL status, sex, and radiosurgical method, indicated that none of these parameters exhibited significant associations with tumor progression [50]. Comparably, in our pooled analysis, the 5-year and 10-year LTC rates were 87% and 80%, respectively, further demonstrating the long-term efficacy of SRS in managing these tumors. Our meta-regression analysis highlighted that age and female sex were significantly correlated with better tumor control outcomes.

This positive correlation between female sex and LTC rates is consistent with findings from a large retrospective study by Kano et al. [9]. Their study revealed that female sex significantly correlates with better tumor control and survival outcomes. They also found that younger age, absence of neurological symptoms, fewer tumors, and higher Karnofsky scores are associated with improved LTC rates. The gender-based differences in outcomes may be attributed to female patients being generally younger at the time of radiosurgery, as reported in several studies [50], and to potential hormonal factors. While multiple studies have identified younger age as a favorable factor for tumor control [9, 12, 13], the positive correlation between age and LTC rate observed in our analyses may be indicative of underlying confounding factors, study heterogeneity, or potential bias rather than a true fundamental association.

Regarding other factors associated with tumor control, hemangioblastomas exhibiting cystic components consistently show worse outcomes and a higher likelihood of progression [9, 20, 23, 31, 32, 51]. SRS is primarily used to treat the mural nodule of the hemangioblastomas and is less effective in reducing the volume of any associated cysts [23].

### Symptom improvement

Most hemangioblastomas detected on imaging are asymptomatic [1, 8]. When neurological symptoms are present, they are most often attributed to the mass effect of the tumor, a peritumoral cyst, or surrounding edema [1, 52, 53]. SRS is a viable option for symptom alleviation in hemangioblastoma patients [9, 12, 36]. A recent study reported symptomatic improvement in 74.9% of lesions across 96.9% of patients following radiotherapy [12]. Consistent with these findings, our analysis revealed a pooled symptom control rate of 84% in patients treated with SRS, indicating its effectiveness in managing symptoms associated with hemangioblastomas.

It is noteworthy that tumor size regression was observed in 28% of cases, while stability was achieved in 59%. These results suggest that although SRS primarily leads to tumor stabilization rather than marked reduction in size, patients typically experience significant clinical improvement. These findings align with previous studies that have investigated symptomatic outcomes [9, 12].

### VHL-Associated vs. Sporadic hemangioblastomas

Hemangioblastomas associated with VHL disease present distinct clinical challenges, characterized by earlier disease onset and a less favorable prognosis due to the development of multiple neoplastic lesions over their lifetime [4]. CNS hemangioblastomas represent the primary cause of mortality in VHL patients [4, 54], accounting for 47.7% of deaths [55].

Prospective natural history studies have elucidated distinctive growth patterns in VHL-associated hemangioblastomas [8, 56]. A prospective study examining 2505 untreated CNS hemangioblastomas demonstrated growth in 49% of lesions over a mean follow-up of 6.9 years, with the predominant pattern being saltatory [72% of growing lesions), characterized by alternating periods of rapid growth and quiescence [8]. This biphasic developmental pattern has been attributed to fluctuating increases in erythropoietin and hypoxia-inducible factors 1α and 2α expression [57].

Given the unpredictable timeline of tumor growth and the inherent risks associated with surgical intervention, treatment is often reserved for symptomatic tumors [47, 49, 56]. SRS can be an effective treatment strategy in cases of multiple hemangioblastomas in VHL patients, considering the additive surgical morbidity associated with repeat resections [12–14].

While the meta-analysis conducted by Pan et al. did not identify a statistically significant difference in tumor progression based on VHL status [50], our results revealed that SRS leads to better tumor control in VHL-associated hemangioblastomas compared to sporadic tumors. There was a significant difference in 3-year and 5-year LTC rates and a near-significant difference in 1-year LTC rate between VHL-associated and sporadic cohorts in our results. Moreover, we observed overall LTC rates of 91% and 85% in VHL and sporadic hemangioblastomas, respectively, but the difference was not significant. Univariate analyses from previous studies have likewise indicated VHL-associated lesions are linked to better control outcomes [19, 30].

Regarding the difference in tumor control rates between VHL-associated and sporadic hemangioblastomas, it is noteworthy that VHL-associated lesions tend to be smaller and asymptomatic at the time of discovery and radiosurgery compared to sporadic lesions [12, 50, 58]. This is primarily because patients with VHL disease are more closely monitored, resulting in earlier tumor detection and often treatment upon radiologic but not clinical progression [1]. Furthermore, VHL-associated hemangioblastomas typically exhibit earlier disease onset, and patients are generally younger at the time of treatment [1, 4]. Several studies have associated younger age [9, 12, 13] and smaller tumor size [12, 13] with improved tumor control rates, which may account for the better outcomes observed in VHL patients.

The understanding of VHL disease biology and pathogenesis has led to the development of systemic therapies targeting HIF-2 transcription. VHL serves as a model for testing belzutifan, the first oral HIF-2 inhibitor approved by the FDA, marking a significant advancement in treating the disease [59]. Future studies may explore the combination of these systemic therapies with radiotherapy, potentially reducing the need for repeated surgeries and minimizing associated morbidity in VHL patients.

### Spinal vs. Intracranial hemangioblastomas

While microsurgical extirpation is the established treatment for symptomatic intramedullary hemangioblastomas [60–63], surgical intervention carries significant inherent risks, particularly for lesions located in the upper cervical spine [64]. Management becomes particularly challenging in VHL patients presenting with multiple lesions [64, 65], thereby establishing SRS as a valuable therapeutic adjuvant [61].

In a retrospective study conducted by Yoo et al., they reported a 5-year LTC rate of 97.4% for spinal hemangioblastomas, which was significantly higher than the 87.8% reported for intracranial hemangioblastomas. Moreover, intracranial hemangioblastomas were reported to remain stable in terms of symptoms, while spinal hemangioblastomas showed significant symptom improvement [12]. In our pooled analysis, the 5-year LTC rate for spinal lesions was 92%, compared to 85% for intracranial lesions, though the difference was not significant. These findings align with prior meta-analyses, which also found no significant differences in control rates between spinal and intracranial hemangioblastomas [50].

### Safety

While SRS is generally considered a safe treatment for hemangioblastomas, potential side effects have been reported. Common adverse events include new-onset neurologic symptoms, radiation necrosis, peritumoral edema, and hydrocephalus [8, 9, 12, 13, 30, 38]. Our meta-analysis demonstrated an adverse event rate of 11%, with a significant positive correlation with marginal and maximum radiation doses and a negative correlation with female sex and the publication year. Improvement in safety outcomes in more recent publications may be attributed to recent advancements in neuroimaging techniques, implementation of the Freiburg protocol for small nodule detection [66, 67], and updated VHL surveillance guidelines [68], enabling earlier intervention when tumors are smaller and asymptomatic.

Our meta-analysis revealed a pooled incidence of adverse radiation effects or radiation necrosis in 9% of patients, demonstrating a positive correlation with the percentage of VHL-associated lesions. Previous investigations have identified VHL presence as a risk factor for adverse radiological events [12]. In cases of tumor progression following upfront SRS treatment, both surgical intervention and additional SRS have proved efficacy in achieving tumor control [13]. Our pooled analysis indicated that 8% of lesions treated with SRS subsequently required surgical resection for symptomatic relief. The requirement for post-SRS resection showed a significant negative correlation with publication year, potentially reflecting improved outcomes through earlier detection and treatment of smaller tumors in more recent studies, as previously discussed.

### Limitations

Our study has several notable limitations. One limitation is that most of the included studies were conducted retrospectively, which may result in selection bias and missing data issues. Additionally, the anatomical location of the lesion, tumor volume, and location to critical structures are factors that often determine the radiosurgical approach and likely the outcome. Notably, dose constraints for critical structures such as the brainstem and visual pathways may impact radiosurgical outcomes. Furthermore, heterogeneity among the studies, particularly in baseline characteristics and treatment modalities, may have contributed to variability in the results. While we attempted to mitigate this through meta-regression and subgroup analyses, some residual variability likely remains. Future prospective, randomized studies with longer follow-up periods are needed to further strengthen the evidence regarding treatment efficacy.

## Conclusion

Our systematic review and meta-analysis demonstrated that SRS is a reasonably effective and safe treatment option for hemangioblastomas, particularly in VHL patients and those with tumors that are often difficult to resect. SRS yields favorable outcomes in tumor control and symptom relief while maintaining low rates of treatment failure and adverse events. Furthermore, our analyses identified several factors that influence radiological and clinical outcomes, including patient demographics, VHL status, and tumor characteristics. These insights enhance patient stratification and risk assessment, enabling more personalized treatment planning.

## Electronic supplementary material

Below is the link to the electronic supplementary material.

Supplementary Material 1

## Data Availability

The data supporting this study’s findings are available from the corresponding author upon reasonable request.
