# Supplementary material for: Stereotactic radiosurgery in the management of central nervous system hemangioblastomas: a systematic review and meta-analysis
Source: Neurosurg Rev. 2025 Mar 17;48(1):303. doi: 10.1007/s10143-025-03454-9 (PMC11911270; doi:10.1007/s10143-025-03454-9)
Supplement: Supplementary file 1 — Supplementary Material 1 [file 10143_2025_3454_MOESM1_ESM.docx]

**Supplementary File**

**Stereotactic Radiosurgery in the Management of Central Nervous System Hemangioblastomas: A Systematic Review and Meta‑Analysis**

Amirhossein Zare^1^, Amirhessam Zare^1^, Alireza Soltani Khaboushan^1^, Bardia Hajikarimloo^2^, Jason P Sheehan^2^

^1^ Department of Neurosurgery, Tehran University of Medical Sciences, Tehran, Iran

^2^ Department of Neurological Surgery, University of Virginia, Charlottesville, VA, USA

***Correspondence:** Jason P Sheehan

Department of Neurological Surgery, University of Virginia, Charlottesville, VA, USA
Email: JPS2F@uvahealth.org

Table 1 The search strategy of electronic databases

| **Database** | **Search Strategy** | **Number** |
| --- | --- | --- |
| PubMed | #1  “Hemangioblastoma”[Mesh] OR “von Hippel-Lindau Disease”[Mesh] OR hemangioblastoma*[tiab] OR haemangioblastoma*[tiab] OR von Hippel-Lindau[tiab] OR Lindau[tiab]  #2   “Radiosurgery”[Mesh] OR radiosurg*[tiab] OR radiation[tiab] OR irradiat*[tiab] OR stereotactic[tiab] OR Gamma Knife[tiab] OR CyberKnife[tiab] OR linear accelerator[tiab] OR LINAC[tiab] OR SRS[tiab] OR FSRT[tiab]  #3 #1 AND #2 | 308 |
| Embase | #1   'hemangioblastoma'/exp OR 'von Hippel Lindau disease'/exp OR hemangioblastoma*:ti,ab OR haemangioblastoma*:ti,ab OR 'von Hippel-Lindau':ti,ab OR Lindau:ti,ab  #2  'srs (stereotactic radiosurgery)'/exp OR 'stereotactic radio-surgery'/exp OR 'stereotaxic radio-surgery'/exp OR 'stereotaxic radiosurgery'/exp  OR radiosurg*:ti,ab OR radiation:ti,ab OR irradiat*:ti,ab OR stereotactic:ti,ab OR 'Gamma Knife':ti,ab OR CyberKnife:ti,ab OR 'linear accelerator':ti,ab OR LINAC:ti,ab OR SRS:ti,ab OR FSRT:ti,ab  #3 #1 AND #2 | 558 |
| WOS | #1 TS= ( “hemangioblastoma*” OR “haemangioblastoma*” OR “von Hippel-Lindau” OR “Lindau” )  #2 TS= ( “radiosurg*” OR “radiation*” OR “irradiat*” OR “stereotactic” OR “Gamma Knife” OR “CyberKnife” OR “linear accelerator” OR “LINAC” OR “SRS” OR “FSRT” )  #3 #1 AND #2 | 416 |
| Scopus | #1 TITLE-ABS-KEY ( “hemangioblastoma*” OR “haemangioblastoma*” OR “von Hippel-Lindau” OR “Lindau” )  #2 TITLE-ABS-KEY ( “radiosurg*” OR “radiation*” OR “irradiat*” OR “stereotactic” OR “Gamma Knife” OR “CyberKnife” OR “linear accelerator” OR “LINAC” OR “SRS” OR “FSRT” )  #3 #1 AND #2 | 726 |
| Cochrane Library | #1 MeSH descriptor: [Hemangioblastoma] explode all trees  #2 MeSH descriptor: [von Hippel-Lindau Disease] explode all trees  #3 (hemangioblastoma* OR haemangioblastoma* OR (von NEXT Hippel NEXT Lindau) OR Lindau):ti,ab,kw  #4 #1 OR #2 OR #3  #5 MeSH descriptor: [Radiosurgery] explode all trees  #6 (radiosurg* OR radiation OR irradiat* OR stereotactic OR (Gamma NEXT Knife) OR CyberKnife OR (linear NEXT accelerator) OR LINAC OR SRS OR FSRT):ti,ab,kw  #7 #5 OR #6  #8 #4 AND #7 | 4 |
| 4 October 2024  Total:2,012, 905 duplicates, 1,107 for screen | |  |

Table 2 Detailed results of meta-regression analyses for different outcomes

| Outcome | Covariate | No. of Studies | Estimate | Estimate SE | P Value | R² | I² | τ² |
| --- | --- | --- | --- | --- | --- | --- | --- | --- |
| 1-Year LTC | Female | 17 | 0.583 | 1.53 | 0.703 | 0 | 39.63 | 0.33 |
|  | Marginal dose | 13 | 0.053 | 0.106 | 0.621 | 0 | 46.12 | 0.37 |
|  | Maximum dose | 11 | 0.029 | 0.042 | 0.489 | 0 | 50 | 0.4 |
|  | Age | 18 | 0.092 | 0.03 | 0.002 | 92.07 | 4.51 | 0.02 |
|  | No. of lesions per patient | 18 | 0.138 | 0.16 | 0.388 | 0 | 38.5 | 0.33 |
|  | Tumor volume | 16 | 0.249 | 0.214 | 0.243 | 11.86 | 35.68 | 0.32 |
|  | VHL associated lesions | 11 | -2.953 | 1.497 | 0.048 | 67.16 | 18.42 | 0.11 |
|  | Publication year | 18 | -0.003 | 0.037 | 0.928 | 0 | 39.29 | 0.36 |
| 3-Year LTC | Female | 17 | 3.201 | 1.179 | 0.007 | 60.46 | 48.23 | 0.17 |
|  | Marginal dose | 13 | 0.021 | 0.095 | 0.824 | 0 | 74.07 | 0.38 |
|  | Maximum dose | 10 | -0.002 | 0.041 | 0.970 | 0 | 63.47 | 0.23 |
|  | Age | 18 | 0.058 | 0.027 | 0.032 | 49.41 | 53.56 | 0.22 |
|  | No. of lesions per patient | 18 | 0.221 | 0.147 | 0.135 | 10.57 | 69.42 | 0.38 |
|  | Tumor volume | 17 | -0.17 | 0.173 | 0.326 | 0 | 72.66 | 0.5 |
|  | VHL associated lesions | 12 | -0.772 | 1.607 | 0.631 | 0 | 75.24 | 0.61 |
|  | Publication year | 18 | 0.039 | 0.031 | 0.205 | 12.71 | 67.96 | 0.37 |
| 5-Year LTC | Female | 18 | 3.638 | 0.947 | < 0.001 | 81.7 | 36.13 | 0.08 |
|  | Marginal dose | 13 | 0.031 | 0.093 | 0.740 | 0 | 76.53 | 0.37 |
|  | Maximum dose | 10 | -0.007 | 0.044 | 0.865 | 0 | 75.23 | 0.35 |
|  | Age | 19 | 0.05 | 0.029 | 0.078 | 32.49 | 65.22 | 0.29 |
|  | No. of lesions per patient | 19 | 0.237 | 0.133 | 0.076 | 22.46 | 71.15 | 0.34 |
|  | Tumor volume | 18 | -0.129 | 0.157 | 0.412 | 0 | 73.31 | 0.42 |
|  | VHL associated lesions | 13 | -0.647 | 1.442 | 0.654 | 0 | 75.37 | 0.49 |
|  | Publication year | 19 | 0.04 | 0.026 | 0.116 | 25.67 | 69.57 | 0.32 |
| Overall LTC | Female | 25 | -0.197 | 1.473 | 0.894 | 0 | 67.03 | 0.41 |
|  | Marginal dose | 17 | 0.061 | 0.054 | 0.259 | 0 | 71.8 | 0.44 |
|  | Maximum dose | 15 | 0.016 | 0.032 | 0.627 | 0 | 66.18 | 0.34 |
|  | Age | 25 | -0.012 | 0.03 | 0.701 | 0 | 65.18 | 0.41 |
|  | No. of lesions per patient | 25 | 0.185 | 0.086 | 0.031 | 27.01 | 58.39 | 0.28 |
|  | Tumor volume | 22 | 0.013 | 0.154 | 0.931 | 0 | 71.38 | 0.44 |
|  | VHL associated lesions | 17 | -1.155 | 0.731 | 0.114 | 26.04 | 59.15 | 0.23 |
|  | Publication year | 25 | -0.006 | 0.022 | 0.785 | 0 | 67.53 | 0.41 |
| 5-Year OS | Female | 9 | 1.459 | 3.028 | 0.630 | 0 | 17.1 | 0.18 |
|  | Marginal dose | 7 | 0.111 | 0.206 | 0.590 | 0 | 50.19 | 0.4 |
|  | Maximum dose | 8 | -0.122 | 0.072 | 0.090 | 30.13 | 24.88 | 0.17 |
|  | Age | 10 | 0.05 | 0.053 | 0.339 | 0.25 | 31.55 | 0.41 |
|  | No. of lesions per patient | 10 | 0.507 | 0.188 | 0.007 | 100 | 0 | 0 |
|  | Tumor volume | 9 | -0.836 | 0.302 | 0.006 | 100 | 0 | 0 |
|  | VHL associated lesions | 7 | 3.425 | 2.581 | 0.184 | 77.64 | 10.81 | 0.09 |
|  | Publication year | 10 | 0.08 | 0.025 | 0.001 | 100 | 0 | 0 |
| Symptom Control | Female | 7 | -1.819 | 2.012 | 0.366 | 33.36 | 0.00 | 0.00 |
|  | Marginal dose | 6 | 0.078 | 0.096 | 0.418 | 0.00 | 0.00 | 0.00 |
|  | Maximum dose | 8 | 0.029 | 0.038 | 0.449 | 59.07 | 0.00 | 0.00 |
|  | Age | 8 | 0.012 | 0.039 | 0.759 | 68.79 | 0.00 | 0.00 |
|  | No. of lesions per patient | 8 | 0.248 | 0.538 | 0.645 | 77.77 | 0.00 | 0.00 |
|  | Tumor volume | 5 | 0.020 | 0.227 | 0.931 | 0.00 | 0.00 | 0.00 |
|  | VHL associated lesions | 4 | -1.559 | 1.825 | 0.393 | 12.47 | 0.00 | 0.00 |
|  | Publication year | 8 | 0.005 | 0.030 | 0.876 | 0.00 | 0.00 | 0.00 |
| Post-SRS Surgical Resection | Female | 16 | -0.369 | 2.259 | 0.87 | 0 | 61.36 | 0.43 |
|  | Marginal dose | 13 | 0.005 | 0.064 | 0.932 | 0 | 59.99 | 0.36 |
|  | Maximum dose | 12 | 0.002 | 0.054 | 0.964 | 0 | 64.96 | 0.48 |
|  | Age | 17 | 0.014 | 0.037 | 0.708 | 0 | 56.84 | 0.39 |
|  | No. of lesions per patient | 17 | -0.276 | 0.116 | 0.017 | 37.23 | 42.42 | 0.2 |
|  | Tumor volume | 16 | 0.181 | 0.161 | 0.262 | 0 | 31.13 | 0.12 |
|  | VHL associated lesions | 12 | -1.6 | 1.2 | 0.182 | 0 | 55.78 | 0.29 |
|  | Publication year | 17 | -0.055 | 0.019 | 0.004 | 59.59 | 32.26 | 0.13 |
| Adverse Event Rate | Female | 21 | -3.503 | 1.515 | 0.021 | 100 | 0 | 0 |
|  | Marginal dose | 16 | 0.134 | 0.056 | 0.017 | 100 | 0 | 0 |
|  | Maximum dose | 13 | 0.069 | 0.032 | 0.033 | 100 | 0 | 0 |
|  | Age | 21 | -0.005 | 0.037 | 0.89 | 0 | 16.41 | 0.12 |
|  | No. of lesions per patient | 21 | 0.008 | 0.129 | 0.949 | 0 | 17.62 | 0.12 |
|  | Tumor volume | 19 | 0.005 | 0.21 | 0.982 | 0 | 20.8 | 0.15 |
|  | VHL associated lesions | 16 | 1.075 | 0.905 | 0.235 | 57.22 | 8.85 | 0.05 |
|  | Publication year | 21 | -0.041 | 0.021 | 0.055 | 80.65 | 3.38 | 0.02 |
| Radiation Necrosis | Female | 13 | -4.582 | 2.234 | 0.04 | 100 | 0 | 0 |
|  | Marginal dose | 9 | 0.125 | 0.075 | 0.094 | 100 | 0 | 0 |
|  | Maximum dose | 8 | 0.022 | 0.054 | 0.681 | 0 | 0 | 0 |
|  | Age | 13 | -0.05 | 0.054 | 0.353 | 15.89 | 12.85 | 0.15 |
|  | No. of lesions per patient | 13 | 0.292 | 0.401 | 0.467 | 0 | 18.89 | 0.24 |
|  | Tumor volume | 11 | -0.086 | 0.36 | 0.81 | 0 | 20.56 | 0.24 |
|  | VHL associated lesions | 11 | 3.795 | 1.564 | 0.015 | 100 | 0 | 0 |
|  | Publication year | 13 | -0.042 | 0.03 | 0.159 | 99.54 | 0.07 | 0 |

Table 3 Detailed results of subgroup analyses

| **Outcome** | **Variable** | **Subgroup** | **No. of Studies** | **No. of Patients/Lesions** | **Pooled Estimate [95% CI]** | **P-Value for Subgroup Difference** |
| --- | --- | --- | --- | --- | --- | --- |
| 1-Year LTC | Marginal dose | <20 | 8 | 869 | 0.96 [0.93-0.98] | 0.57 |
|  |  | ≥20 | 4 | 139 | 0.97 [0.93-0.99] |  |
| 1-Year LTC | Maximum dose | <30 | 6 | 752 | 0.96 [0.91-0.98] | 0.86 |
|  |  | ≥30 | 5 | 285 | 0.96 [0.93-0.98] |  |
| 1-Year LTC | Region | East Asia | 5 | 761 | 0.97 [0.94-0.99] | 0.25 |
|  |  | North America | 9 | 425 | 0.94 [0.91-0.96] |  |
|  |  | Other | 4 | 133 | 0.96 [0.91-0.99] |  |
| 1-Year LTC | Modality | Gamma Knife | 9 | 966 | 0.97 [0.95-0.99] | 0.01 |
|  |  | Cyber Knife | 4 | 81 | 0.91 [0.82-0.96] |  |
| 1-Year LTC | Mean tumor volume | <2 | 9 | 855 | 0.96 [0.93-0.98] | 0.85 |
|  |  | ≥2 | 7 | 357 | 0.96 [0.92-0.98] |  |
| 1-Year LTC | Year of publication | >2010 | 9 | 982 | 0.97 [0.95-0.98] | 0.05 |
|  |  | ≤2010 | 9 | 337 | 0.94 [0.90-0.96] |  |
| 3-Year LTC | Marginal dose | <20 | 7 | 855 | 0.87 [0.78-0.93] | 0.81 |
|  |  | ≥20 | 5 | 221 | 0.86 [0.80-0.90] |  |
| 3-Year LTC | Maximum dose | <30 | 5 | 738 | 0.90 [0.85-0.94] | 0.71 |
|  |  | ≥30 | 5 | 285 | 0.88 [0.78-0.94] |  |
| 3-Year LTC | Region | East Asia | 5 | 761 | 0.91 [0.85-0.95] | 0.67 |
|  |  | North America | 10 | 507 | 0.89 [0.83-0.93] |  |
|  |  | Other | 3 | 119 | 0.84 [0.50-0.96] |  |
| 3-Year LTC | Modality | Gamma Knife | 9 | 966 | 0.90 [0.81-0.95] | 0.94 |
|  |  | Cyber Knife | 3 | 67 | 0.90 [0.80-0.95] |  |
| 3-Year LTC | Mean tumor volume | <2 | 10 | 937 | 0.91 [0.87-0.94] | 0.39 |
|  |  | ≥2 | 7 | 357 | 0.87 [0.75-0.94] |  |
| 3-Year LTC | Year of publication | >2010 | 8 | 968 | 0.91 [0.86-0.94] | 0.24 |
|  |  | ≤2010 | 10 | 419 | 0.86 [0.78-0.92] |  |
| 5-Year LTC | Marginal dose | <20 | 7 | 855 | 0.84 [0.73-0.91] | 0.95 |
|  |  | ≥20 | 5 | 221 | 0.84 [0.78-0.89] |  |
| 5-Year LTC | Maximum dose | <30 | 5 | 738 | 0.87 [0.82-0.91] | 0.90 |
|  |  | ≥30 | 5 | 285 | 0.86 [0.72-0.94] |  |
| 5-Year LTC | Region | East Asia | 5 | 761 | 0.90 [0.78-0.96] | 0.85 |
|  |  | North America | 11 | 642 | 0.88 [0.82-0.91] |  |
|  |  | Other | 3 | 119 | 0.84 [0.50-0.96] |  |
| 5-Year LTC | Modality | Gamma Knife | 9 | 966 | 0.88 [0.78-0.94] | 0.54 |
|  |  | Cyber Knife | 4 | 202 | 0.91 [0.86-0.94] |  |
| 5-Year LTC | Mean tumor volume | <2 | 11 | 1072 | 0.89 [0.85-0.92] | 0.56 |
|  |  | ≥2 | 7 | 357 | 0.86 [0.74-0.93] |  |
| 5-Year LTC | Year of publication | >2010 | 9 | 1103 | 0.90 [0.85-0.93] | 0.13 |
|  |  | ≤2010 | 10 | 419 | 0.84 [0.74-0.90] |  |
| 10-Year LTC | Maximum dose | <30 | 2 | 561 | 0.72 [0.52-0.86] | 0.22 |
|  |  | ≥30 | 2 | 143 | 0.90 [0.59-0.98] |  |
| 10-Year LTC | Year of publication | >2010 | 2 | 614 | 0.79 [0.76-0.82] | 0.77 |
|  |  | ≤2010 | 2 | 90 | 0.85 [0.27-0.99] |  |
| Overall LTC | Marginal dose | <20 | 9 | 869 | 0.86 [0.77-0.92] | 0.20 |
|  |  | ≥20 | 7 | 252 | 0.91 [0.87-0.94] |  |
| Overall LTC | Maximum dose | <30 | 9 | 818 | 0.85 [0.78-0.90] | 0.53 |
|  |  | ≥30 | 6 | 200 | 0.89 [0.74-0.96] |  |
| Overall LTC | modality | Gamma Knife | 10 | 938 | 0.92 [0.85-0.95] | 0.41 |
|  |  | LINAC | 3 | 79 | 0.88 [0.78-0.94] |  |
|  |  | Cyber Knife | 7 | 241 | 0.87 [0.82-0.90] |  |
| Overall LTC | Region | East Asia | 5 | 752 | 0.92 [0.87-0.95] | 0.28 |
|  |  | North America | 13 | 664 | 0.87 [0.80-0.91] |  |
|  |  | Other | 7 | 176 | 0.92 [0.84-0.96] |  |
| Overall LTC | Mean tumor volume | <2 | 15 | 1217 | 0.90 [0.85-0.93] | 0.83 |
|  |  | ≥2 | 7 | 343 | 0.89 [0.79-0.94] |  |
| Overall LTC | Year of publication | >2010 | 13 | 1178 | 0.89 [0.85-0.92] | 0.82 |
|  |  | ≤2010 | 12 | 414 | 0.90 [0.81-0.95] |  |
| Tumor Progression | Marginal dose | <20 | 9 | 869 | 0.14 [0.08-0.23] | 0.20 |
|  |  | ≥20 | 7 | 252 | 0.09 [0.06-0.13] |  |
| Tumor Progression | Maximum dose | <30 | 9 | 818 | 0.15 [0.10-0.22] | 0.53 |
|  |  | ≥30 | 6 | 200 | 0.11 [0.04-0.26] |  |
| Tumor Progression | modality | Gamma Knife | 10 | 938 | 0.08 [0.05-0.15] | 0.41 |
|  |  | LINAC | 3 | 79 | 0.12 [0.06-0.22] |  |
|  |  | Cyber Knife | 7 | 241 | 0.13 [0.10-0.18] |  |
| Tumor Progression | Region | East Asia | 5 | 752 | 0.08 [0.05-0.13] | 0.28 |
|  |  | North America | 13 | 664 | 0.13 [0.09-0.20] |  |
|  |  | Other | 7 | 176 | 0.08 [0.04-0.16] |  |
| Tumor Progression | Mean tumor volume | <2 | 15 | 1217 | 0.10 [0.07-0.15] | 0.83 |
|  |  | ≥2 | 7 | 343 | 0.11 [0.06-0.21] |  |
| Tumor Progression | Year of publication | >2010 | 13 | 1178 | 0.11 [0.08-0.15] | 0.82 |
|  |  | ≤2010 | 12 | 414 | 0.10 [0.05-0.19] |  |
| Tumor Regression | Marginal dose | <20 | 5 | 638 | 0.32 [0.24-0.42] | 0.61 |
|  |  | ≥20 | 5 | 198 | 0.39 [0.18-0.65] |  |
| Tumor Regression | Maximum dose | <30 | 6 | 734 | 0.30 [0.22-0.40] | 0.95 |
|  |  | ≥30 | 3 | 65 | 0.31 [0.10-0.65] |  |
| Tumor Regression | modality | Gamma Knife | 6 | 636 | 0.30 [0.16-0.48] | 0.82 |
|  |  | LINAC | 2 | 43 | 0.17 [0.02-0.65] |  |
|  |  | Cyber Knife | 3 | 150 | 0.32 [0.07-0.76] |  |
| Tumor Regression | Region | East Asia | 3 | 571 | 0.41 [0.18-0.68] | 0.55 |
|  |  | North America | 8 | 448 | 0.24 [0.13-0.41] |  |
|  |  | Other | 5 | 144 | 0.28 [0.12-0.51] |  |
| Tumor Regression | Mean tumor volume | <2 | 12 | 1081 | 0.29 [0.18-0.42] | 0.03 |
|  |  | ≥2 | 2 | 64 | 0.12 [0.06-0.23] |  |
| Tumor Regression | Year of publication | >2010 | 7 | 894 | 0.22 [0.13-0.35] | 0.23 |
|  |  | ≤2010 | 9 | 269 | 0.35 [0.20-0.54] |  |
| 5-Year Overall Survival | Marginal dose | <20 | 4 | 240 | 0.86 [0.74-0.93] | 0.62 |
|  |  | ≥20 | 2 | 38 | 0.92 [0.47-0.99] |  |
| 5-Year Overall Survival | Maximum dose | <30 | 4 | 255 | 0.95 [0.84-0.98] | 0.02 |
|  |  | ≥30 | 4 | 55 | 0.78 [0.65-0.87] |  |
| 5-Year Overall Survival | Region | East Asia | 2 | 199 | 0.89 [0.83-0.93] | 0.54 |
|  |  | North America | 5 | 132 | 0.95 [0.80-0.99] |  |
|  |  | Other | 3 | 29 | 0.87 [0.67-0.96] |  |
| 5-Year Overall Survival | Modality | Gamma Knife | 5 | 251 | 0.85 [0.75-0.91] | 0.06 |
|  |  | Cyber Knife | 3 | 68 | 0.97 [0.86-1.00] |  |
| 5-Year Overall Survival | Mean tumor volume | <2 | 7 | 308 | 0.91 [0.86-0.94] | 0.72 |
|  |  | ≥2 | 2 | 42 | 0.87 [0.43-0.98] |  |
| Post-SRS Surgical Resection | Marginal dose | <20 | 8 | 800 | 0.08 [0.05-0.14] | 0.51 |
|  |  | ≥20 | 4 | 133 | 0.12 [0.05-0.28] |  |
| Post-SRS Surgical Resection | Maximum dose | <30 | 5 | 743 | 0.07 [0.05-0.11] | 0.44 |
|  |  | ≥30 | 7 | 229 | 0.11 [0.04-0.23] |  |
| Post-SRS Surgical Resection | modality | Gamma Knife | 9 | 849 | 0.08 [0.04-0.16] | 0.31 |
|  |  | Cyber Knife | 3 | 178 | 0.04 [0.01-0.14] |  |
|  |  | LINAC | 2 | 59 | 0.12 [0.06-0.23] |  |
| Post-SRS Surgical Resection | Region | East Asia | 3 | 660 | 0.05 [0.03-0.07] | 0.04 |
|  |  | North America | 10 | 603 | 0.08 [0.05-0.11] |  |
|  |  | Other | 4 | 72 | 0.15 [0.07-0.31] |  |
| Post-SRS Surgical Resection | Mean tumor volume | <2 | 9 | 1008 | 0.06 [0.04-0.08] | 0.28 |
|  |  | ≥2 | 7 | 316 | 0.09 [0.05-0.14] |  |
| Post-SRS Surgical Resection | Year of publication | >2010 | 9 | 1054 | 0.06 [0.04-0.08] | 0.04 |
|  |  | ≤2010 | 8 | 281 | 0.11 [0.06-0.20] |  |
| Radiation Necrosis | Marginal dose | ≥20 | 6 | 99 | 0.14 [0.08-0.24] | 0.07 |
|  |  | <20 | 3 | 46 | 0.03 [0.01-0.14] |  |
| Radiation Necrosis | Maximum dose | <30 | 5 | 83 | 0.03 [0.01-0.10] | 0.37 |
|  |  | ≥30 | 3 | 27 | 0.07 [0.02-0.25] |  |
| Radiation Necrosis | modality | LINAC | 3 | 45 | 0.03 [0.01-0.15] | 0.69 |
|  |  | Gamma Knife | 4 | 43 | 0.07 [0.02-0.21] |  |
|  |  | Cyber Knife | 4 | 78 | 0.05 [0.02-0.13] |  |
| Radiation Necrosis | Region | North America | 7 | 147 | 0.10 [0.05-0.19] | 0.43 |
|  |  | Other | 6 | 60 | 0.06 [0.02-0.16] |  |
| Radiation Necrosis | Mean tumor volume | <2 | 9 | 153 | 0.10 [0.06-0.18] | 0.43 |
|  |  | ≥2 | 2 | 34 | 0.05 [0.01-0.23] |  |
| Radiation Necrosis | Year of publication | >2010 | 8 | 121 | 0.06 [0.03-0.12] | 0.07 |
|  |  | ≤2010 | 5 | 86 | 0.15 [0.08-0.25] |  |
| Worsened Symptoms | Maximum dose | <30 | 6 | 94 | 0.17 [0.10-0.28] | 0.48 |
|  |  | ≥30 | 2 | 62 | 0.07 [0.01-0.53] |  |
| Worsened Symptoms | modality | LINAC | 2 | 31 | 0.19 [0.09-0.37] | 0.53 |
|  |  | Gamma Knife | 2 | 62 | 0.07 [0.01-0.53] |  |
|  |  | Cyber Knife | 3 | 43 | 0.09 [0.02-0.34] |  |
| Worsened Symptoms | Mean tumor volume | <2 | 3 | 57 | 0.17 [0.08-0.32] | 0.86 |
|  |  | ≥2 | 2 | 49 | 0.18 [0.10-0.32] |  |
| Worsened Symptoms | Year of publication | >2010 | 3 | 47 | 0.16 [0.06-0.34] | 0.91 |
|  |  | ≤2010 | 5 | 109 | 0.17 [0.10-0.26] |  |
| Adverse Radiation Events | Marginal dose | <20 | 9 | 309 | 0.09 [0.06-0.14] | 0.04 |
|  |  | ≥20 | 6 | 105 | 0.18 [0.11-0.28] |  |
| Adverse Radiation Events | Maximum dose | <30 | 8 | 316 | 0.06 [0.04-0.10] | 0.01 |
|  |  | ≥30 | 5 | 61 | 0.17 [0.10-0.29] |  |
| Adverse Radiation Events | modality | Gamma Knife | 8 | 278 | 0.11 [0.07-0.18] | 0.47 |
|  |  | LINAC | 3 | 45 | 0.05 [0.01-0.17] |  |
|  |  | Cyber Knife | 5 | 96 | 0.09 [0.04-0.17] |  |
| Adverse Radiation Events | Region | East Asia | 4 | 229 | 0.11 [0.06-0.21] | 0.84 |
|  |  | North America | 10 | 212 | 0.11 [0.07-0.19] |  |
|  |  | Other | 7 | 80 | 0.09 [0.04-0.18] |  |
| Adverse Radiation Events | Mean tumor volume | <2 | 13 | 400 | 0.11 [0.07-0.16] | 0.72 |
|  |  | ≥2 | 6 | 101 | 0.09 [0.05-0.18] |  |
| Adverse Radiation Events | Year of publication | >2010 | 11 | 361 | 0.08 [0.06-0.12] | 0.07 |
|  |  | ≤2010 | 10 | 160 | 0.15 [0.09-0.23] |  |


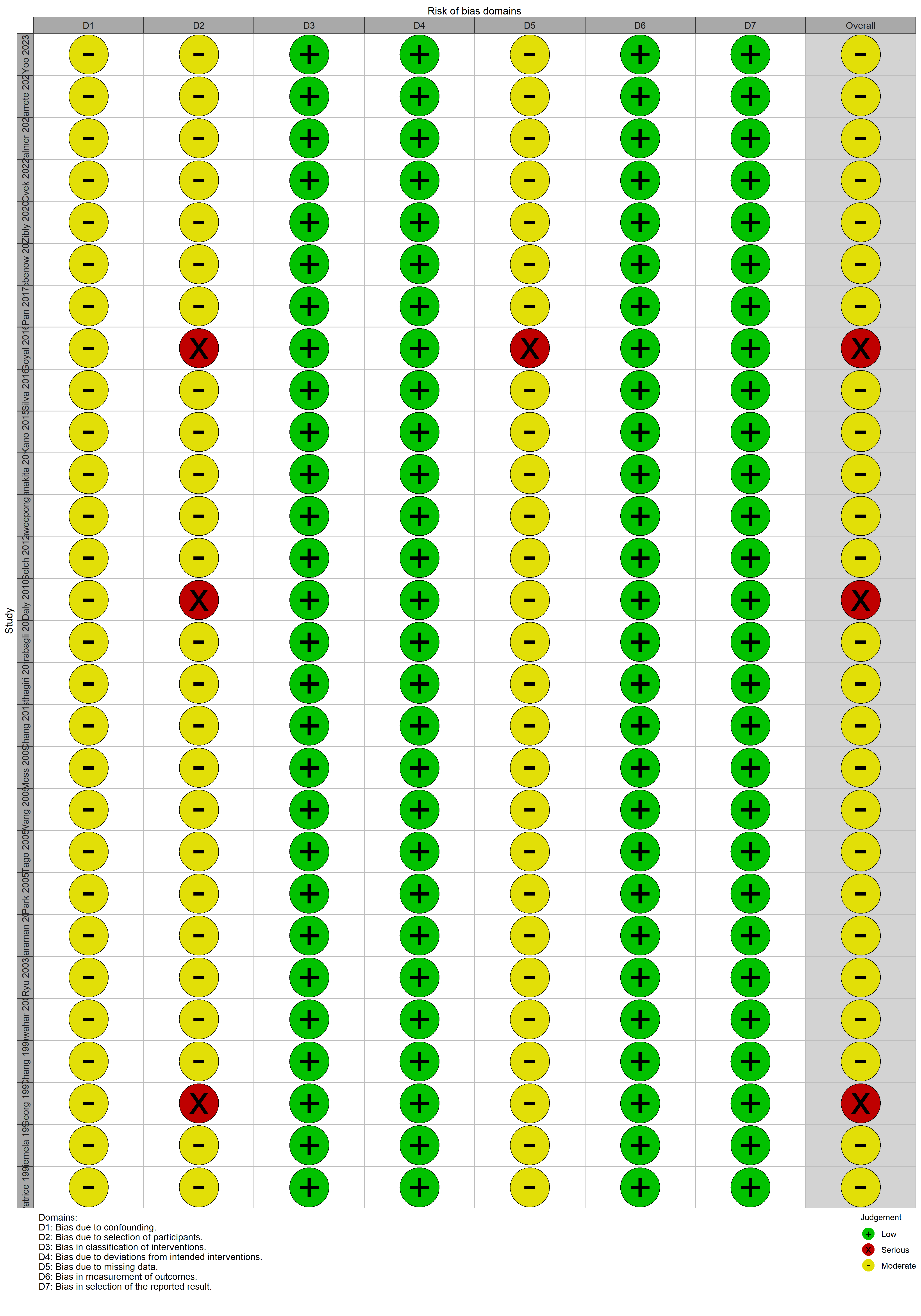


Figure 1 Risk of bias assessment based on the ROBINS-I tool





Figure 2 Sensitivity analysis of 1-year LTC

f



Figure 3 Sensitivity analysis of 3-year LTC





Figure 4 Sensitivity analysis of 5-year LTC





Figure 5 Sensitivity analysis of 10-year LTC





Figure 6 Sensitivity analysis of overall LTC





Figure 7 Sensitivity analysis of stable tumor rate





Figure 8 Sensitivity analysis of tumor regression





Figure 9 Sensitivity analysis of 5-year OS





Figure 10 Sensitivity analysis of symptom control





Figure 11 Sensitivity analysis of post-SRS surgical resection





Figure 12 Sensitivity analysis of adverse event rate





Figure 13 Sensitivity analysis of radiation necrosis


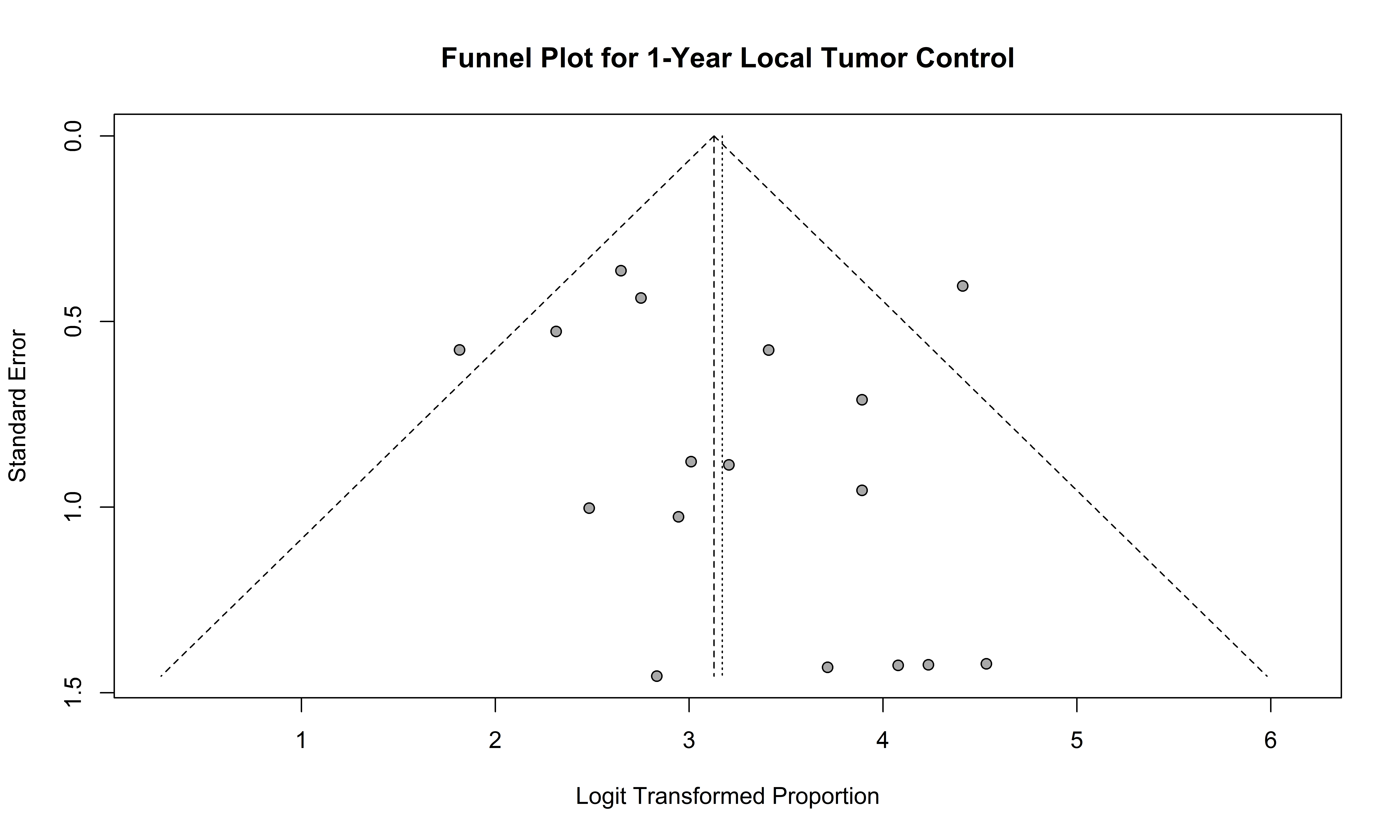


Figure 14 Funnel plot of 1-year LTC


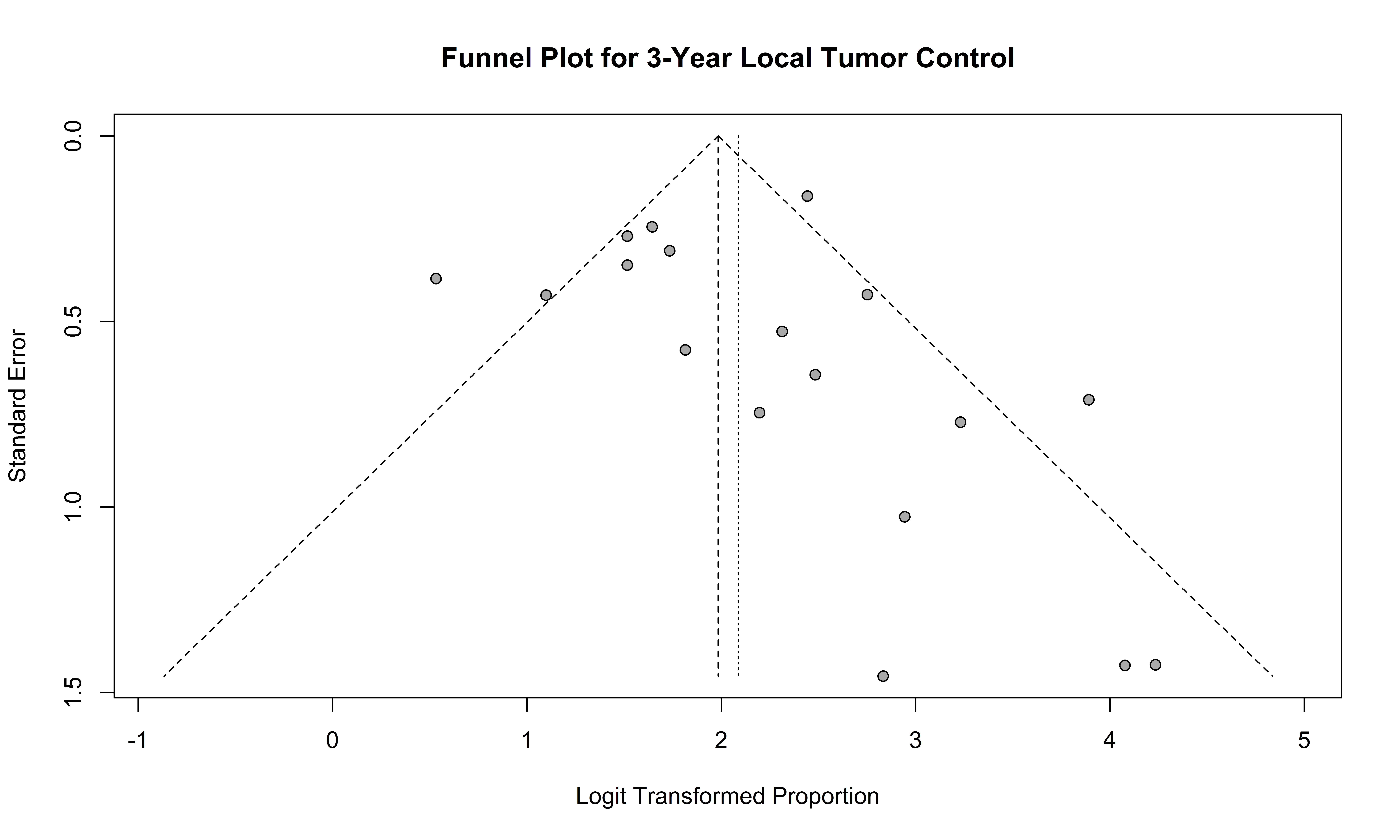


Figure 15 Funnel plot of 3-year LTC


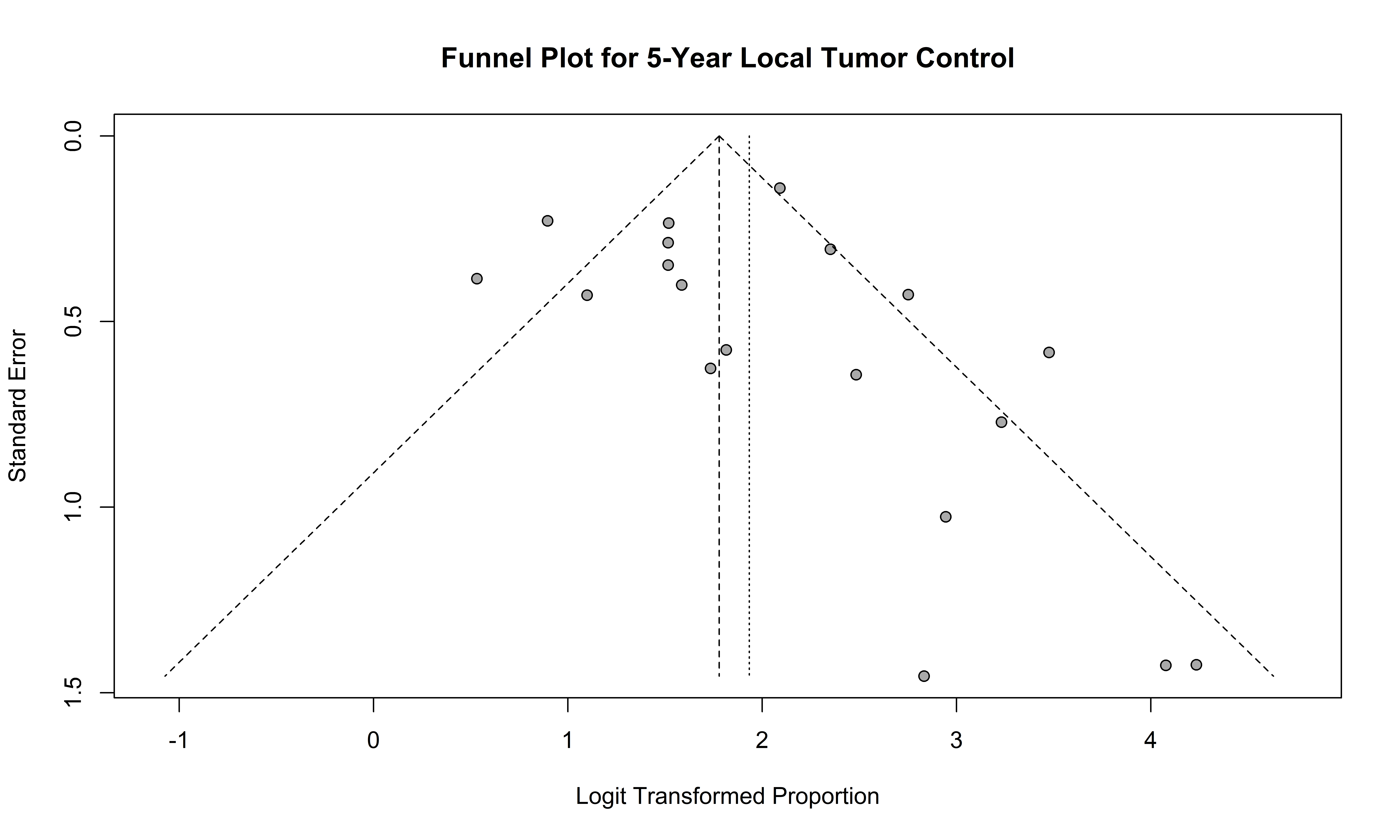


Figure 16 Funnel plot of 5-year LTC


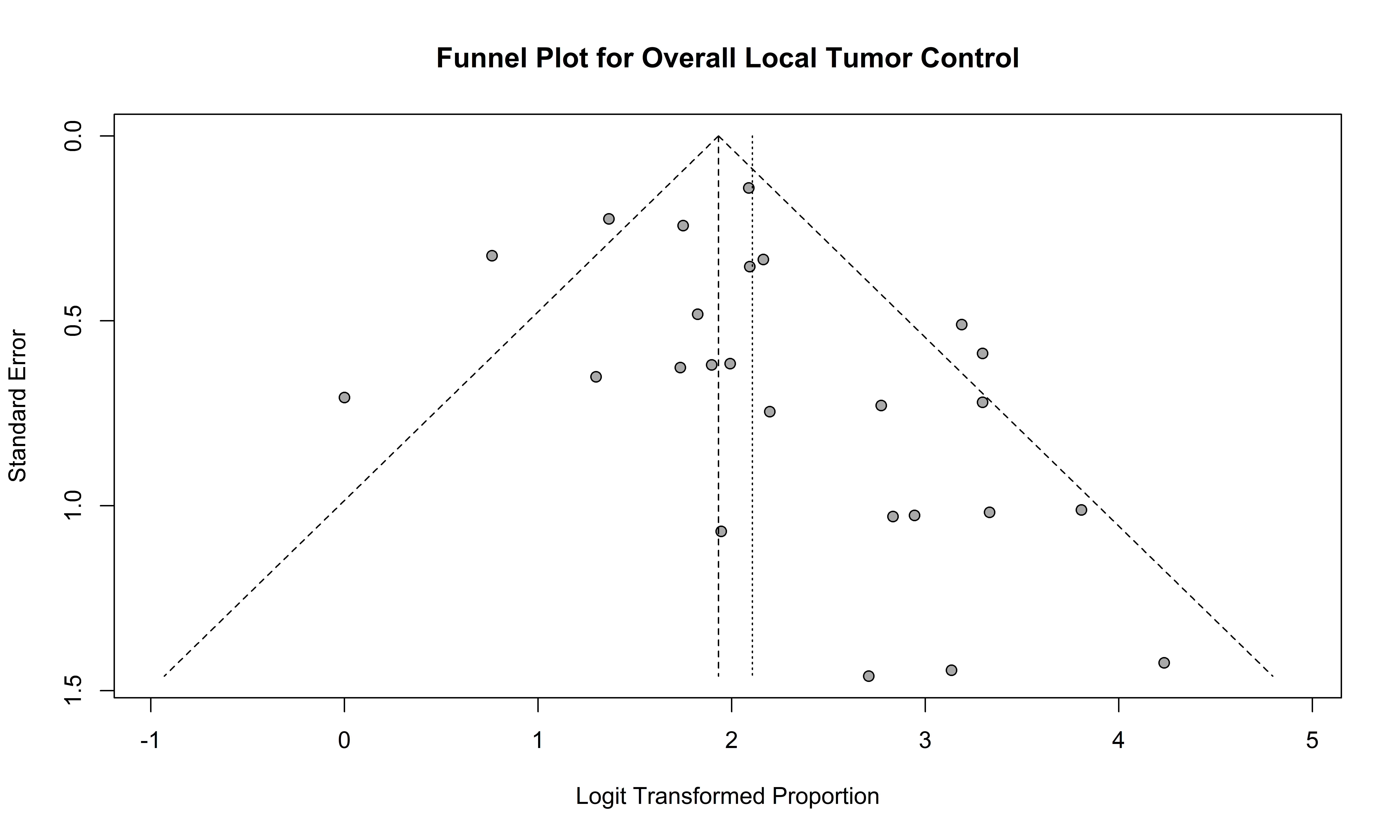


Figure 17 Funnel plot of overall LTC


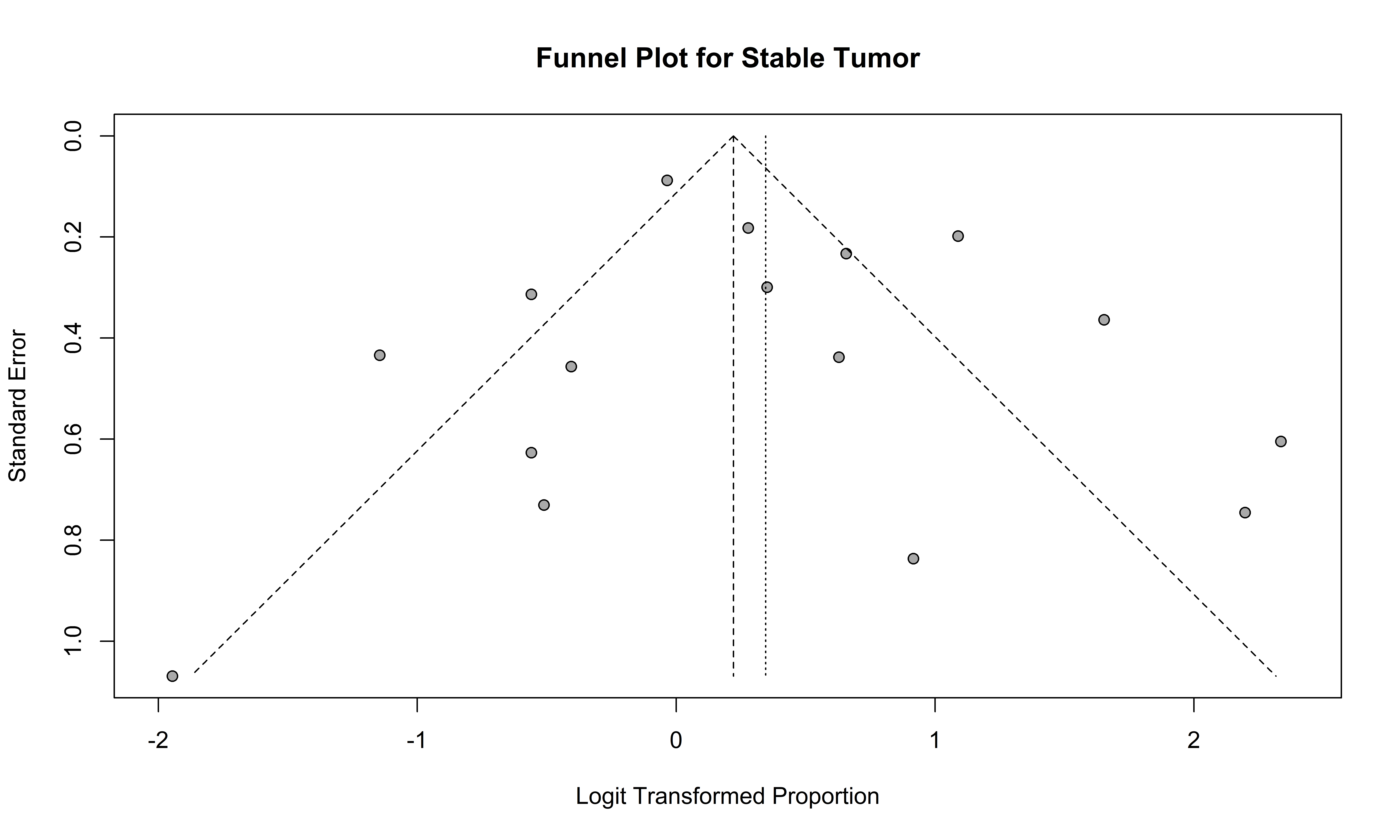


Figure 18 Funnel plot of stable tumor rate


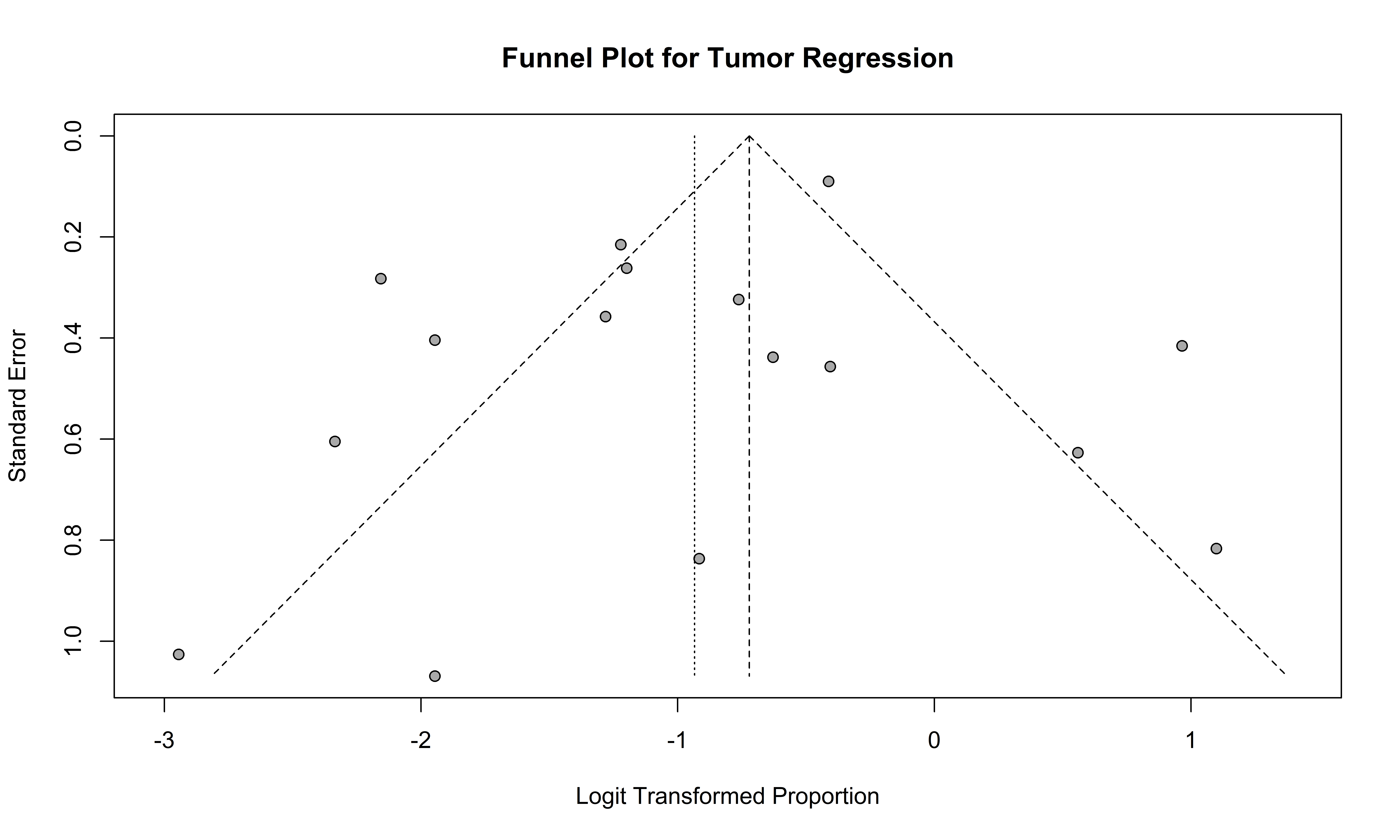


Figure 19 Funnel plot of tumor regression


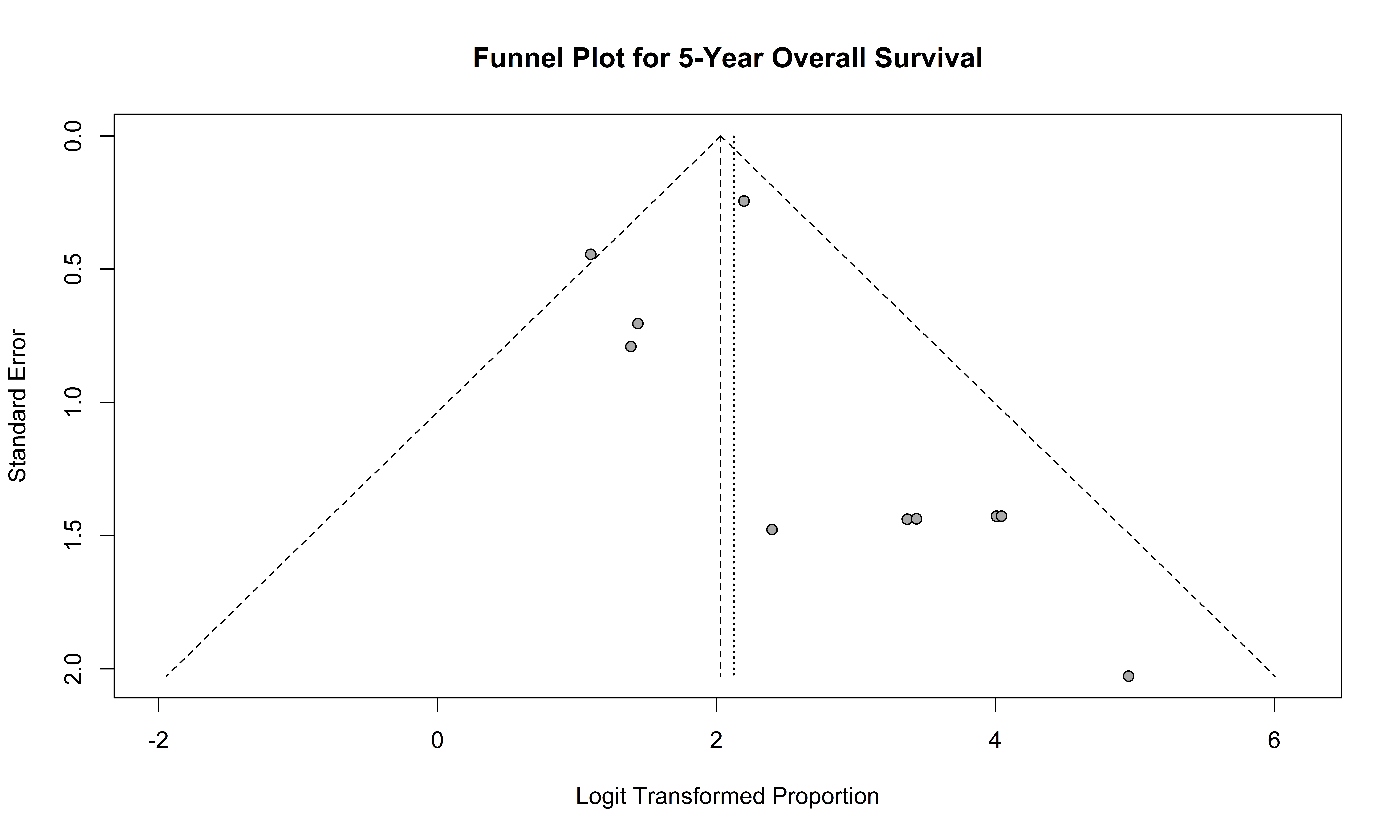


Figure 20 Funnel plot of 5-year OS


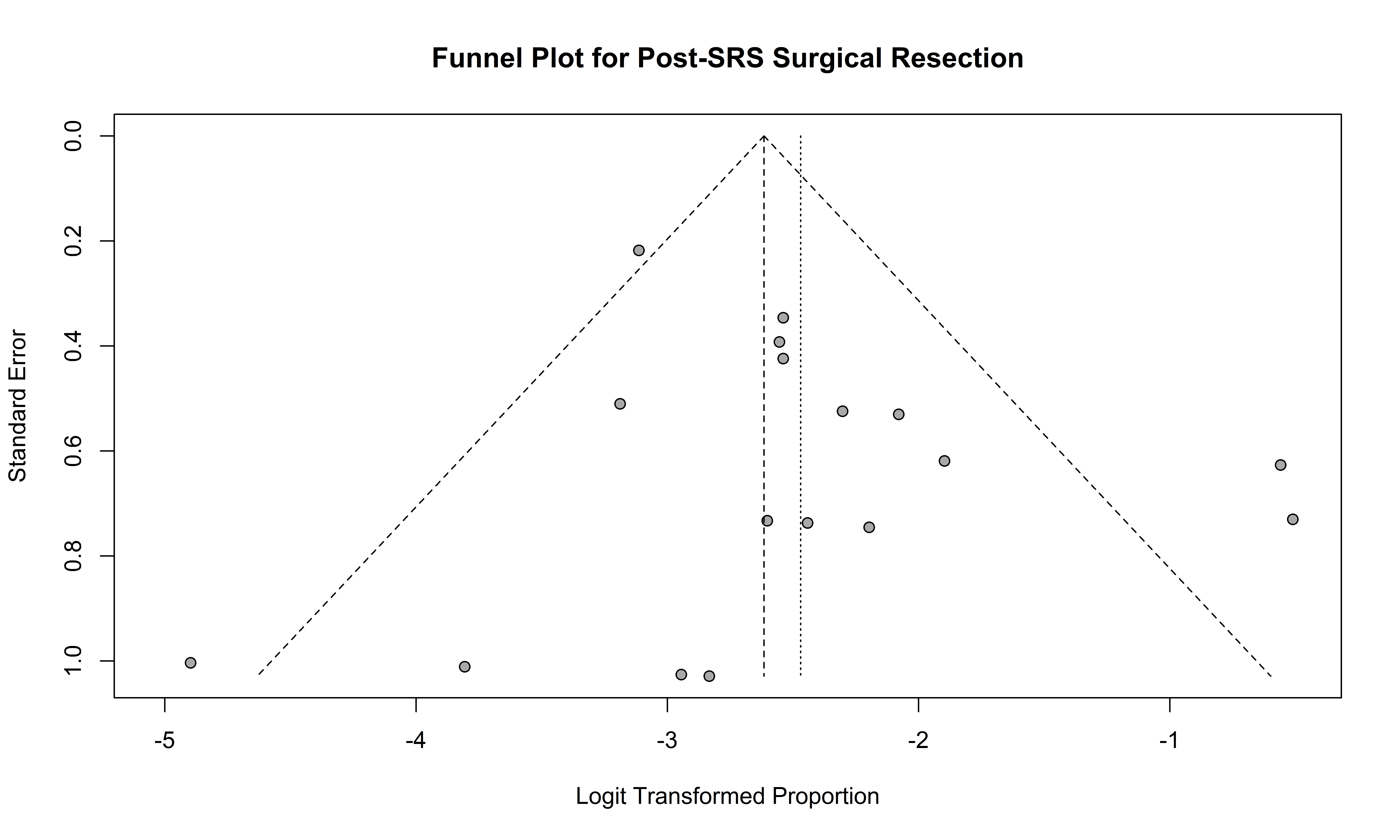


Figure 21 Funnel plot of post-SRS surgical resection


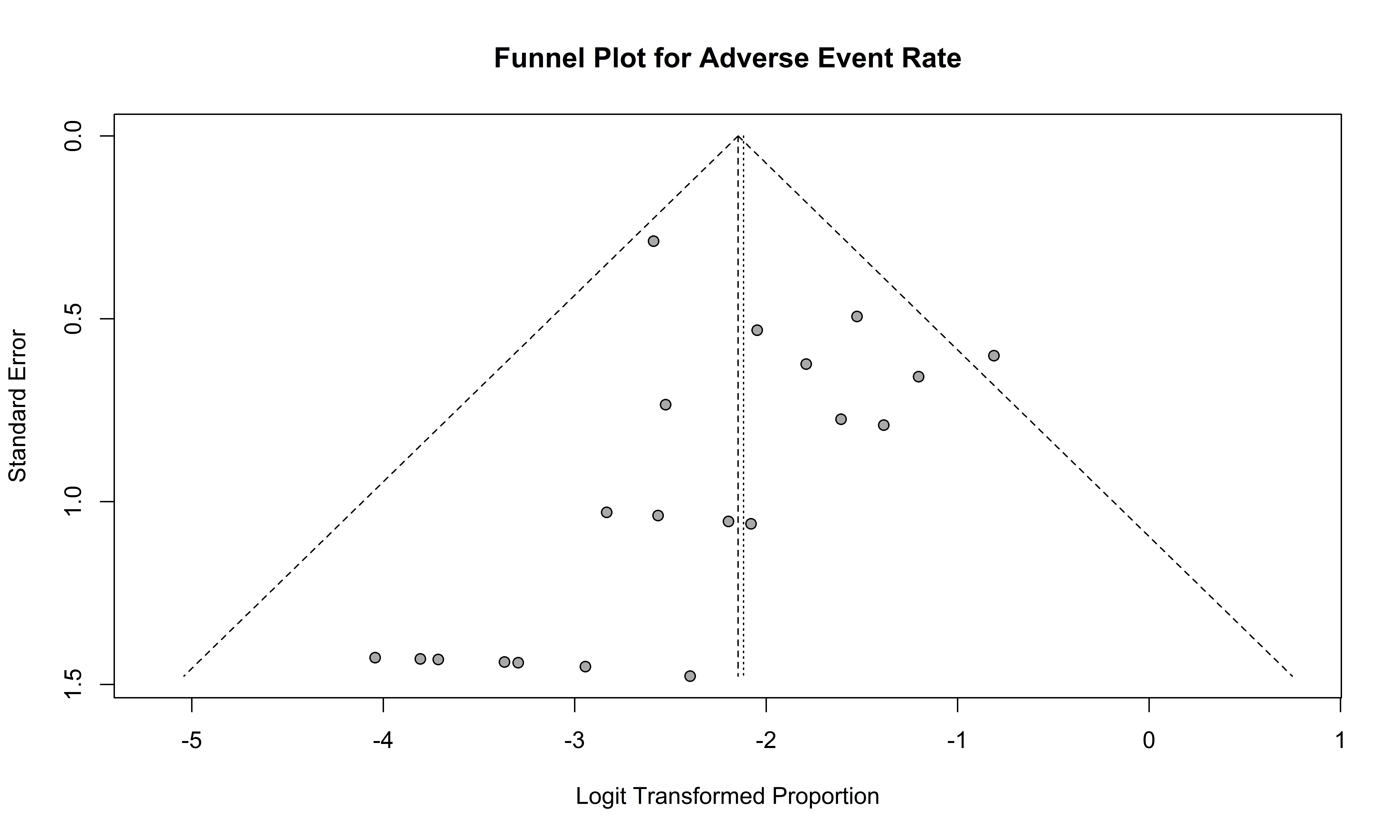


Figure 22 Funnel plot of adverse event rate


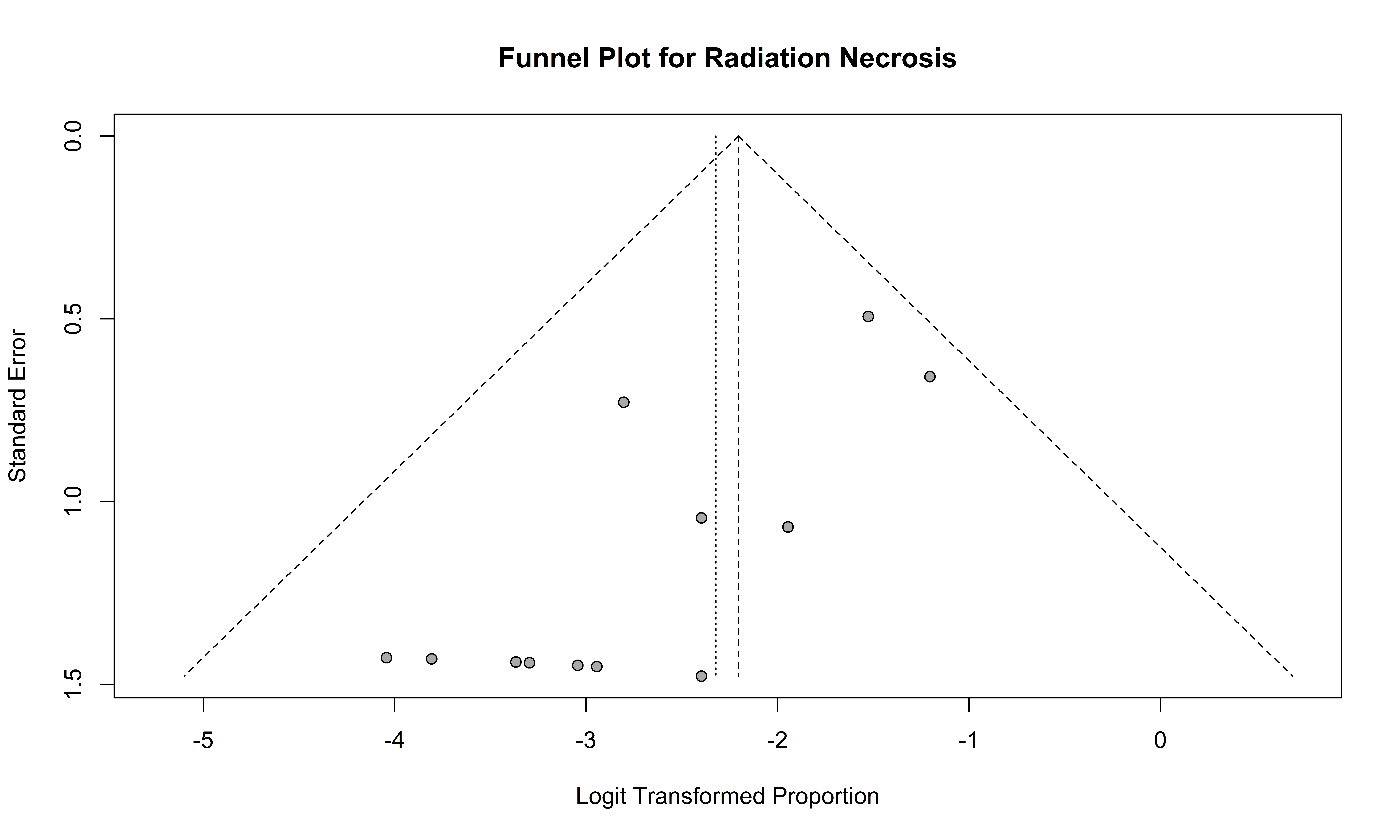


Figure 23 Funnel plot of radiation necrosis


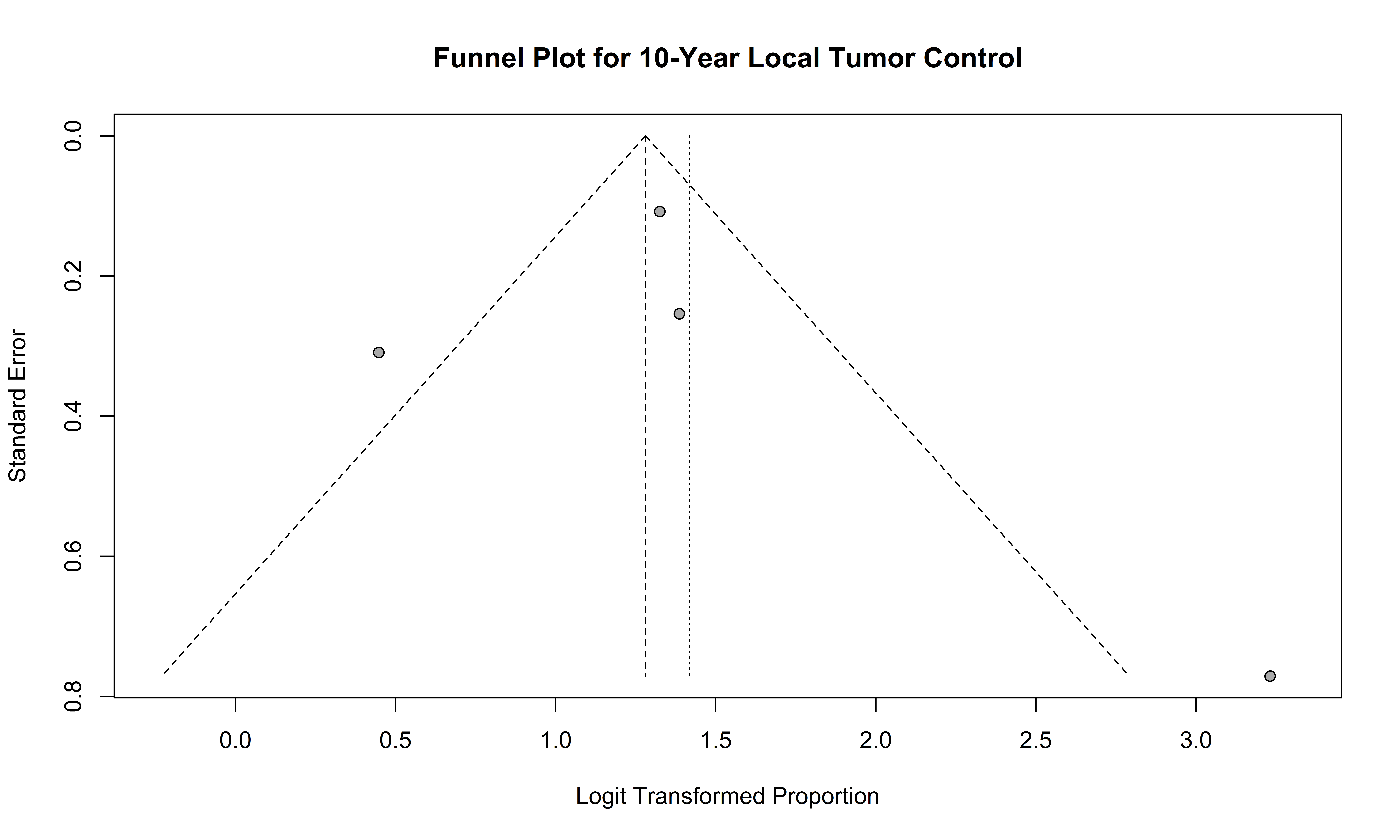


Figure 24 Funnel plot of 10-year LTC


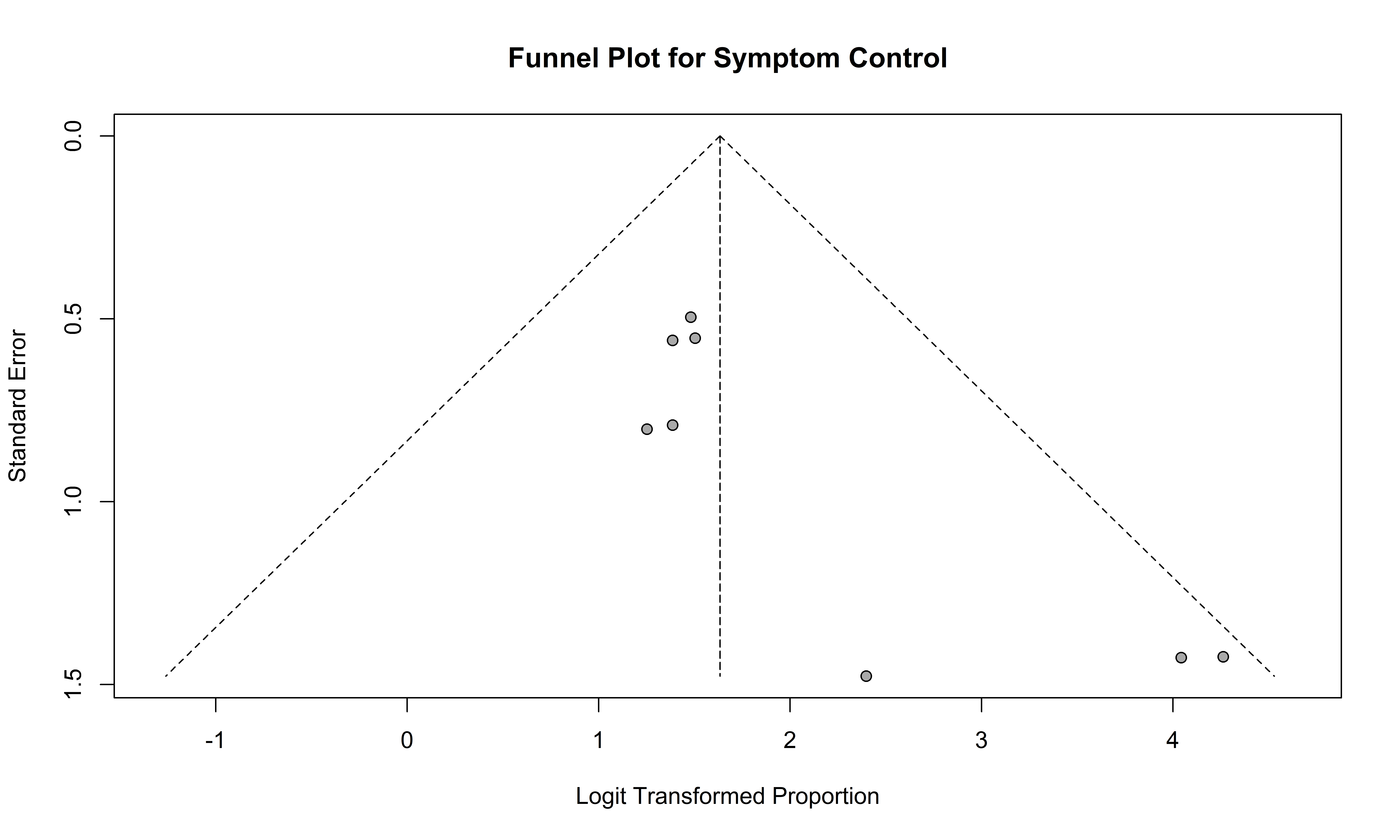


Figure 25 Funnel plot of symptom control
